# 3D‐Printed Architected Anisotropic Channels for Ultrafast Solar‐Driven Interfacial Evaporation via Localized Thermal Management and Water Layer Structuring

**DOI:** 10.1002/advs.202520694

**Published:** 2026-01-28

**Authors:** Sijia Sun, Dong Jiang, Hengsong Zheng, Shuai Zhang, Ziyuan Cheng, Changtong Mei, Dan Tian, Shilong Yang, Yusuke Yamauchi, Mingzhu Pan

**Affiliations:** ^1^ College of Materials Science and Engineering Co‐Innovation Centre of Efficient Processing and Utilization of Forest Resources Nanjing Forestry University Nanjing China; ^2^ Australian Institute for Bioengineering and Nanotechnology (AIBN) The University of Queensland Brisbane QLD Australia; ^3^ Advanced Analysis and Testing Center Nanjing Forestry University Nanjing China; ^4^ Department of Materials Process Engineering Graduate School of Engineering Nagoya University Nagoya Japan; ^5^ Department of Chemical and Biomolecular Engineering Yonsei University Seoul South Korea

**Keywords:** 3D‐Print, anisotropic channels, solar‐driven interfacial evaporation, thermal management, water layer structuring

## Abstract

Efficient solar‐driven interfacial evaporation requires coordinated photon absorption, heat confinement, and directional water delivery, yet current directional evaporators rarely achieve materials‐level anisotropy to regulate photon‐phonon‐water coupling. Here, we report a 3D‐printed anisotropic channel architecture (a‐BTCG) that co‐engineers directional geometry with preferential alignment of Ti_3_O_5_ nanoparticles, boron nitride (BN) nanosheets, and chitosan to form an integrated transport framework. The a‐BTCG delivers a high evaporation rate of 5.43 kg m^−2^ h^−1^ under 1 sun and maintains stable performance for over 200 h in 20 wt.% NaCl, enabled by fast water flux (1.13 × 10^−2^ µm^3^ s^−1^) and enhanced in‐plane thermal conductivity (2.73 W m^−1^ K^−1^). Mechanistic investigations reveal that aligned BN establishes continuous phonon‐guided thermal pathways for heat localization, while the Ti_3_O_5_–BN hybrids enhance broadband absorption via reduced reflectance and multireflection in oriented channels. Chitosan mediates interfacial water structuring, lowers effective evaporation enthalpy, and sustains salt‐resistant replenishment. The combined structural and materials‐level anisotropy, therefore, overcomes conventional trade‐offs in light absorption, heat dissipation, and water supply. This work demonstrates the potential of 3D printing–assisted alignment engineering for high‐performance solar evaporators and provides a generalizable platform for advanced desalination and environmental thermal‐management technologies.

## Graphical abstract

A 3D‐printed anisotropic channel evaporator integrates directional geometry with aligned titanium oxide, boron nitride, and chitosan networks to coordinate photon absorption, heat localization, and water transport. Phonon‐guided thermal pathways and interfacial water structuring enable ultrahigh evaporation rates and long‐term salt‐resistant solar desalination.

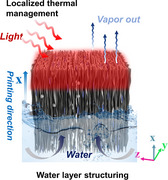

## 1 Introduction

The pursuit of light as a means of harnessing and converting energy has never ceased, driving fundamental advances in solar‐thermal technologies [1, 2, 3]. Solar‐driven interfacial evaporation, as a key implementation of solar‐thermal technologies, enables water vapor generation below the boiling point and steam production at or above it [4, 5, 6, 7]. It has emerged as a promising strategy in sustainable applications, including seawater desalination [8], wastewater treatment [9], clean water generation [10], thermal energy harvesting [11], as well as resource recycling [12]. Unlike conventional bottom heating and volumetric heating strategies, the recently developed solar‐driven interfacial evaporation approach improves heat localization at the liquid surface and has successfully achieved an evaporation efficiency of ∼90% under reduced optical concentrations [5]. Despite remarkable progress in photothermal materials and device architectures, several fundamental challenges continue to limit performance, including insufficient heat localization, poor regulation of interfacial water chemistry, and salt accumulation under high‐salinity conditions. These persistent bottlenecks highlight the urgent need for architected materials capable of simultaneously optimizing heat transport, interfacial thermodynamics, and water management.

Recent studies have shown that directional porous architectures, constructed via freeze‐casting, templating, wood‐inspired scaffolds, or additive manufacturing, can substantially enhance evaporation efficiency by providing aligned transport pathways that reduce tortuosity, accelerate brine drainage, and partially confine heat near the air‐water interface [13, 14]. Directionally aligned channels represent an important step toward engineered transport [7, 15, 16, 17, 18]. However, the anisotropy in these systems arises predominantly from geometric orientation, while the underlying material microstructure and chemical environment remain essentially isotropic and weakly regulated. Consequently, the thermal conduction network remains discontinuous, interfacial water molecules retain bulk‐like enthalpy, and salt management still relies primarily on passive natural convection. Therefore, although structural anisotropy improves mass and heat transport, material‐level anisotropy and chemical microenvironment engineering, both essential for advancing solar‐thermal energy utilization, remain largely underexplored.

To overcome these limitations, an effective evaporator must integrate directionally aligned channels with a multifunctional composite composition, enabling synergistic control over light absorption, heat transport, and interfacial water behavior within the same vertically oriented porous framework. Within such a composite system, the photothermal component plays a central role in harvesting solar energy. Among the available options, Titanium suboxides (TSOs, Ti_n_O_2n‐1_) have attracted increasing attention due to their vacancy‐induced electronic structures, strong broadband absorption, and cost‐effective photothermal conversion [19, 20]. Their tunable optical and electronic properties make TSOs, particularly *λ*‐Ti_3_O_5_, highly competitive candidates for efficient vapor generation [21, 22]. Efficient thermal regulation is equally essential for solar‐driven interfacial evaporation. 2D Boron nitride nanosheet (BN) possesses intrinsically anisotropic phonon transport and outstanding chemical stability, enabling directional thermal conduction and effective heat localization near the air‐liquid interface. When incorporated into aligned porous structures, BN can facilitate in‐plane heat spreading while minimizing downward heat loss and simultaneously contribute to capillary‐driven water delivery due to its sheet‐like morphology [23, 24]. In addition to photothermal and thermal‐regulation elements, sustaining continuous water replenishment requires a hydrophilic matrix capable of mediating interfacial water chemistry. Chitosan (CS) provides strong water wettability and forms abundant hydrogen‐bond interactions, which help maintain hydration and modulate the interfacial water network, an essential factor for reducing the effective evaporation enthalpy [5, 25, 26]. Together, Ti_3_O_5_, BN, and CS offer complementary functionalities ideally suited for constructing a hierarchically organized composite evaporator, in which light harvesting, phonon‐guided heat management, and interfacial water regulation operate cooperatively within aligned vertical channels.

A key challenge, however, lies in how to assemble these components into a hierarchically oriented architecture in which BN nanosheets form directional conduction pathways, Ti_3_O_5_ nanoparticles remain uniformly dispersed for effective light absorption, and the CS network creates a regulated interfacial chemical environment. 3D printing, particularly direct ink writing (DIW), offers a unique solution [18, 25, 26, 27, 28, 29, 30, 31, 32]. By finely tuning ink rheology to balance shear‐thinning, viscoelasticity, and thixotropy, extrusion printing generates controlled shear fields that can induce partial preferential alignment of BN nanosheets [18, 24, 25, 26, 27, 28, 29, 30, 31, 32, 33, 34, 35, 36, 37]. This enables the creation of continuous, low‐resistance phonon pathways that cannot be achieved through geometric channel alignment alone. Our previous work demonstrated the feasibility of printing‐induced hierarchical alignment in MXene‐CNF systems, validating DIW as a powerful platform for constructing multi‐scale anisotropic architectures [30]. Based on the above considerations, such an advanced nanoarchitecture hybrid system can be constructed, where Ti_3_O_5_ nanoparticles serve as solar harvesting materials and oriented BN function as thermal regulators and water supply pathways.

Herein, we report a multi‐material, multi‐scale anisotropic evaporator (a‐BTCG, where a indicates aligned, B represents boron nitride nanosheets, T denotes Ti_3_O_5_, C means chitosan, and G denotes glutaraldehyde) composed of directionally organized BN nanosheets, broadband Ti_3_O_5_ nanoparticles, and a hydrophilic CS matrix. The printing process enables both geometric anisotropy and material‐level preferential orientation, giving rise to continuous in‐plane phonon pathways, efficient broadband light absorption, and a chemically regulated interfacial water environment. These coupled effects establish a photon‐phonon‐water interaction network, which enhances heat localization, lowers the effective evaporation enthalpy, and enables rapid thermal response. Benefiting from this multi‐scale anisotropic design, a‐BTCG achieves a high evaporation rate of 5.43 kg m^−2^ h^−1^ under 1 sun, anisotropic thermal conductivity of 2.73 W m^−1^ K^−1^, and exceptional long‐term stability, maintaining performance for over 200 h in 20 wt.% NaCl. Moreover, the integrated thermal pathways endow the system with fast thermal sensing capability, underscoring its multifunctional nature. This work demonstrates a materials‐structure‐chemistry integrated design paradigm, in which 3D printing serves as an enabling platform for orchestrating anisotropic transport and interfacial thermodynamic regulation, offering a promising route for next‐generation solar desalination and thermal management technologies.

## 2 Results and Discussion

### 2.1 Design Principles for Architected Anisotropic Channels

**FIGURE 1:**
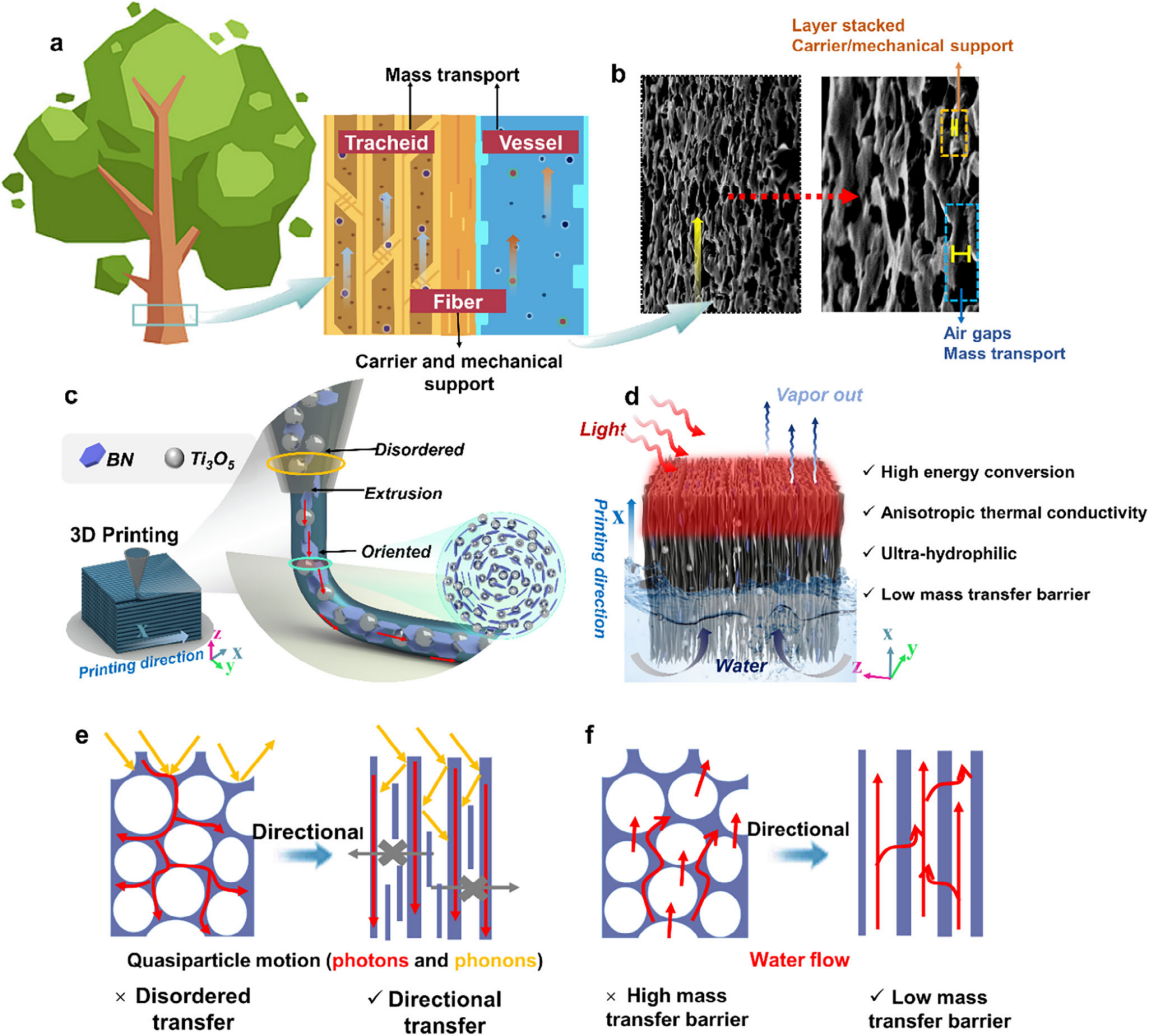
Bioinspired dual‐anisotropy design of the BTCG solar‐driven interfacial evaporation. (a) Directed water and nutrient transport in natural wood is enabled by vertically aligned vessels and tracheids. (b) SEM morphology of the biomimetic aerogel showing a wood‐like cellular channel structure that supports mechanical stability and directional mass transport. (c) Schematic illustration of 3D‐printing‐induced directional assembly. (d) Working principle of the a‐BTCG evaporator under solar‐driven interfacial evaporation. (e) Photon and phonon transport pathways, showing that aligned architectures enhance multireflection, light harvesting, and in‐plane thermal conduction. (f) Water‐transport behavior in disordered versus aligned channels, where anisotropic channels reduce tortuosity and enable rapid, continuous capillary flow.

The microstructure of natural wood consists of a network of horizontally stratified cells (tracheid) and vertically interconnected channels (vessels) that enable nutrients to be transported upwards by capillary action. Beyond transport, the fibrous matrix also offers structural integrity and functional adaptability (Figure 1a,b). Motivated by this hierarchical efficiency, we aimed to replicate and adapt these features into a synthetic system for solar‐driven desalination. To this end, we designed a biomimetic aerogel framework featuring vertically aligned hydrophilic channels, fabricated via 3D printing using GA‐CS as the matrix and BN as the structural scaffold (Figure 1c,d). Ti_3_O_5_ nanoparticles are strategically incorporated within the BN channel walls to serve as photothermal conversion units. The rationales behind this architecture are (i) Ti_3_O_5_, integrated into the oriented walls, enables broadband solar absorption and enhances in‐channel light refraction, thereby reducing reflection losses and improving photothermal conversion efficiency (Figure 1e); (ii) the aligned BN induce quantum confinement of phonons, facilitating directional thermal transport and suppressing heat dissipation via anisotropic conductivity; (iii) the superhydrophilic CS matrix, together with low‐curvature pore channels, accelerates capillary‐driven water transport while minimizing mass transfer resistance (Figure 1f). Compared to conventional porous materials, this synergistically engineered structure exhibits superior photothermal and water‐handling performance, as evidenced by benchmark comparisons (Tables S1 and S2).

### 2.2 Fabrication and Characterization of a‐BTCG

To realize the 3D‐printed anisotropic framework, we first developed printable ink by systematically engineering the composition and rheology. Details of the synthesis process and basic characteristics are provided in the Method section and Supporting Information (Figures S1–S14). The ink was formulated by dispersing Ti_3_O_5_ nanoparticles and BN into a CS matrix, with GA as a chemical crosslinker. Through careful tuning of component ratios, we achieved a viscoelastic ink with pronounced shear‐thinning behavior and high structural fidelity (Figure S4), enabling smooth extrusion and directional alignment during printing.

**FIGURE 2:**
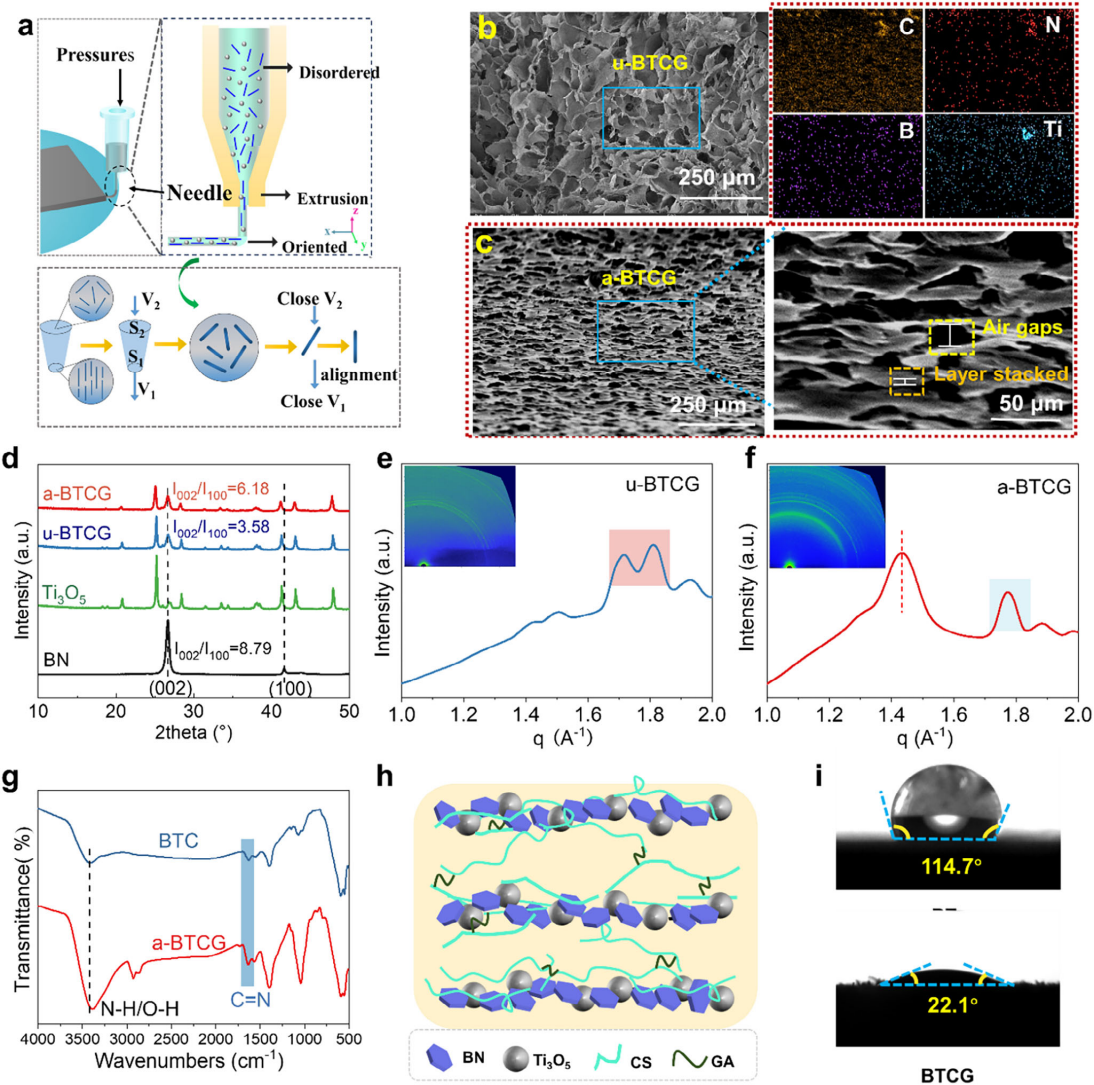
Construction and characterization. (a) Schematic illustration of the 3D‐printing process, where shear alignment during extrusion orients BN nanosheets and constructs a materials‐level anisotropic architecture. (b) SEM and EDS mapping of u‐BTCG, showing a random porous network. (c) SEM images of aligned a‐BTCG, presenting vertically oriented channels formed by layer‐by‐layer printing, with stacked lamellae and interconnected air gaps. (d) XRD patterns comparing structural features of a‐BTCG, u‐BTCG, and Ti_3_O_5_. (e–f) 2D SAXS patterns and corresponding azimuthal intensity plots of u‐BTCG and a‐BTCG, confirming enhanced BN orientation induced by 3D printing. (g) FTIR spectra of BTC and a‐BTCG. h) Schematic diagram of the composite a‐BTCG network showing the distribution of BN nanosheets, Ti_3_O_5_ nanoparticles, CS chains, and GA crosslinkers. (i) Water‐contact‐angle measurements before and after CS introduction, demonstrating the transition to a superhydrophilic surface beneficial for solar‐driven interfacial evaporation.

The 3D printing process effectively induced orientation of the anisotropic BN. Under extrusion, high shear forces along the Z‐axis aligned BN edges perpendicular to the nozzle cross‐section, forming vertically ordered channels upon deposition (Figure 2a) [37]. The alignment mechanism follows the flow velocity continuity equation (S_1_V_1_ = S_2_V_2_, S_1_<S_2_, V_1_>V_2_ leads to the orientation of anisotropic BN), and the corresponding shear stress distribution along the nozzle radius is given by:

(1) $$\tau =\left(\Delta P/2L\right)\;\times \;r$$

where *L* represents the length of the nozzle, *r* denotes the radial distance, and ∆*P* is the applied pressure difference. The equation indicates that the maximum shear stress (τ_m__a__x_) is exerted at the nozzle wall (r = R) and decreases linearly toward the center of the nozzle. Within the nozzle, low shear stress near the core leads to plug flow, while higher shear stress near the walls induces differential flow. This gradient creates a shear flow field, known as the differential flow region, where anisotropic particles such as BN experience sufficient torque to reorient along the flow direction [38]. The cross‐sectional scanning electron microscopy SEM images clearly show that the pore morphology evolves from a randomly distributed, disordered network in the unaligned BTCG (u‐BTCG) (Figure 2b) to a‐BTCG with a well‐defined, directionally aligned lamellar architecture after 3D printing (Figure 2c). The stacked layers and the interlayer air gaps formed during printing provide continuous pathways that facilitate efficient mass transport and quasiparticle propagation. In addition, the EDS elemental mapping shows a uniform spatial distribution of B and N from BN nanosheets and well‐dispersed Ti from Ti_3_O_5_ nanoparticles throughout the polymer matrix. This confirms that the multicomponent system forms a homogeneous hybrid architecture without phase segregation.

XRD was used to confirm the structure of the composites. The peaks at 26.9° and 41.6° correspond to the (002) and (100) crystal planes of BN, respectively, and the orientation of BN can be determined by calculating the peak intensity ratio (Figure 2d). The intensity of the (002) peak of BN is much larger than (100) due to the length of BN nanosheets being much larger than the thickness. Thus, the BN nanosheets are spontaneously aligned along the horizontal direction under gravity with an *I_002_*/*I_001_* ratio of 8.79. The u‐BTCG is difficult for BN to spontaneously achieve orientation due to the obstruction of the Ti_3_O_5_ particles and the CS gel network, and the *I_002_*/*I_001_* ratio is significantly reduced to 3.58. However, BN has a better oriented structure as the *I_002_*/*I_001_* ratio rises to 6.18 under the shear force during 3D printing [39]. The orientation of BN was further quantitatively characterized by small‐angle X‐ray scattering (SAXS). Different from the patterns shown in Figure 2e that suggest the disorder arrangement of BN, the distinct arcs observed in the 2D scattering patterns in Figure 2f indicate a high degree of horizontal orientation of BN via the 3D printing technology. The ordered structure is shown on a 1D scattering curve and allows direct determination of the long‐period size of the system through the Bragg equation:

(2) d=2π/q

where *d* is the periods or dimensions of ordered structures, and *q* is the scattering vector. A clear peak intensity of the BNorientated structure appears at the scattering vector 1.4 A^−1^ (Figure 2f), indicating a long‐range‐orientated structure (ordered and laminated BN layers). Ordered structures of smaller size are indicated at 1.7–1.8 A^−1^, where the structure of the system exhibits homogenization (reduced number of peaks) due to the directional arrangement of the BN compared to the disordered system (Figure 2e).

The chemical structure of the printed composites was validated by fourier transform infrared (FTIR) spectroscapy and X‐ray photoelectron spectriscopy (XPS). Figure 2g presents the FTIR spectra of BTC (structure without GA) and a‐BTCG, highlighting the chemical changes associated with GA‐mediated crosslinking. Upon introduction of glutaraldehyde, the a‐BTCG spectrum shows a distinct imine C = N stretching band appearing near 1650 cm^−1^, which partially overlaps with the amide I region of chitosan, signifying the condensation reaction between GA aldehydes and chitosan ‐NH_2_ groups. A weak peak at ∼1731 cm^−^^1^ arises from residual unreacted GA aldehyde (C = O stretching). The broad N‐H/O‐H stretching band of pristine chitosan at 3352 shifts to 3338 cm^−1^ in BTC and further to 3310 cm^−1^ in a‐BTCG. This progressive redshift reflects the strengthening and reorganization of the hydrogen‐bonding network caused by the introduction of BN/Ti_3_O_5_ surfaces [38]. XPS peaks at 288.6/399, 401.4, and 402.3 eV correspond to the synthesized C = N and a small amount of unreacted amide (C = N) and amino (N ‐ H), respectively, verifying the formation of imine bonds and electrostatic interactions (Figure S3) [40]. The a‐BTCG framework is constructed through the homogeneous integration of BN nanosheets, Ti_3_O_5_ nanoparticles, CS, and GA, as schematically illustrated in Figure 2h. This component‐level organization forms a continuous and interconnected hybrid network, which is stabilized through a combination of covalent cross‐linking and non‐covalent interactions, which collectively maintain the mechanical integrity and uniform dispersion of all components. This robust integrated architecture supports efficient heat management and mass transport during solar‐driven interfacial evaporation. Additionally, contact angle measurements confirmed the inherent hydrophilicity of the CS matrix (Figure 2i), essential for capillary‐driven water transport. The superhydrophilic behavior originates primarily from the chitosan matrix, which contains abundant ─OH and ─NH_2_ groups capable of forming strong hydrogen bonding with water. During ink preparation and GA crosslinking, these polar functional groups remain exposed, enabling rapid spreading and complete wetting of water on the BTCG surface. In addition, the incorporation of BN nanosheets and the vertically aligned microchannels produced by 3D printing facilitates fast capillary uptake and continuous water replenishment. These combined chemical and structural features result in the persistent superhydrophilicity observed in a‐BTCG. Collectively, this integrated synthesis strategy, combining component‐level tuning, ink rheology control, and directional structure alignment, enables the fabrication of a structurally ordered and chemically robust BTCG system tailored for efficient solar‐driven interfacial evaporation.

**FIGURE 3:**
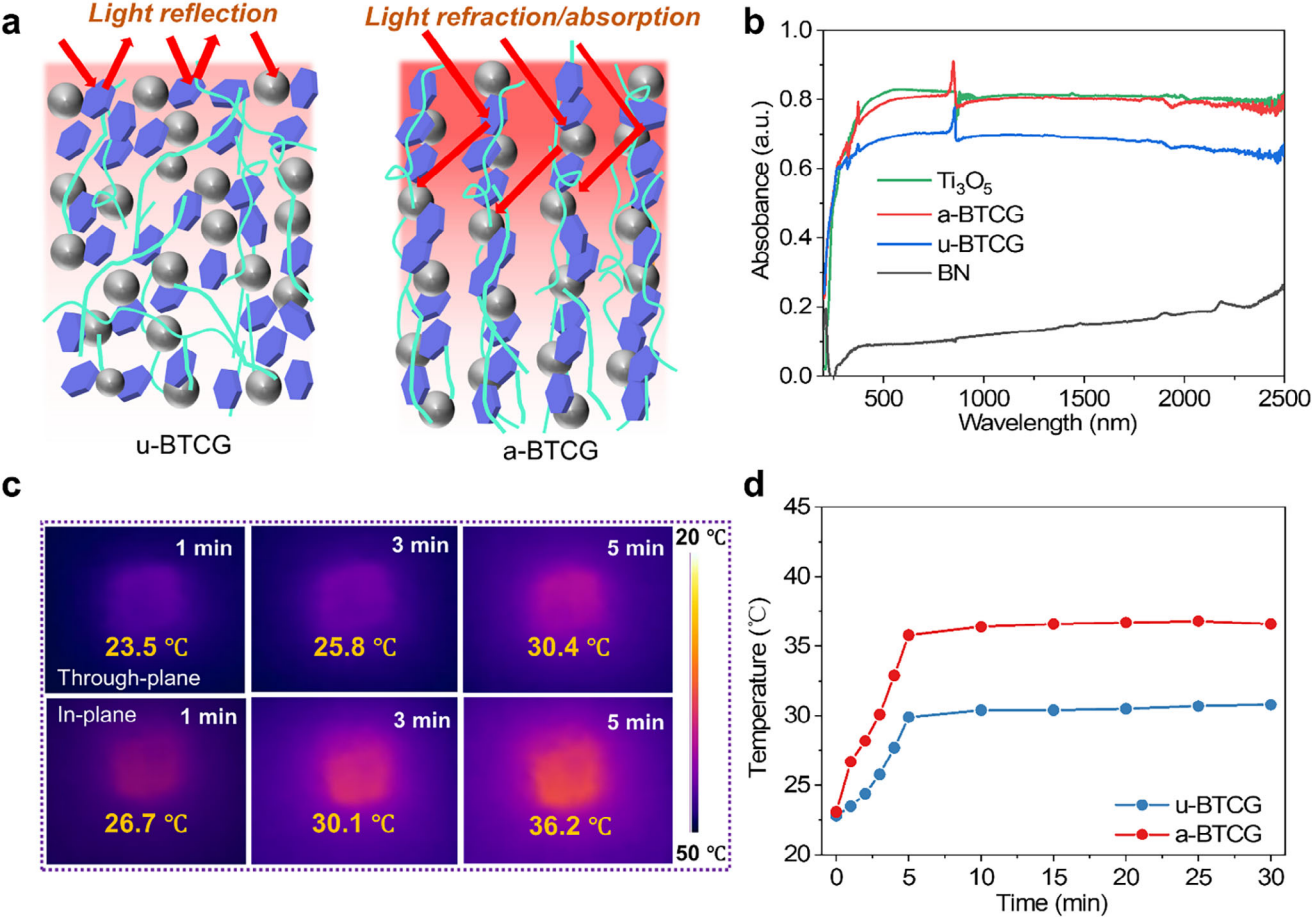
Photothermal Conversion and Thermal Response Performance. (a) Schematic illustration of photon transport pathways in u‐BTCG (left) and a‐BTCG (right), highlighting differences in light reflection, refraction, and absorption. (b) UV–Vis–NIR absorptance spectra (200–2500 nm) of Ti_3_O_5_ nanoparticles, a‐BTCG, u‐BTCG, and BN nanosheets, demonstrating enhanced broadband absorption in the aligned system. (c) Infrared thermal images of the a‐BTCG evaporator floating on seawater under 1‐sun illumination, comparing through‐plane and in‐plane heating behaviors. (d) Surface temperature evolution of a‐BTCG and u‐BTCG under continuous illumination, showing accelerated and higher steady‐state temperature rise in the aligned architecture.

Photothermal Conversion and Thermal Response Performance. Efficient solar‐driven interfacial evaporation critically depends on two key factors: strong light‐to‐heat conversion and rapid thermal energy transport. Materials that can effectively harvest solar energy and facilitate directional heat flow are essential for minimizing energy loss and maximizing interfacial water vapor generation. The vertically aligned BN network within the a‐BTCG structure plays a pivotal role in managing light and thermal energy. The aligned architecture promotes multiple internal reflections along the vertical channels, effectively extending the photon residence time and enhancing light trapping. (Figure 3a) To more comprehensively assess the solar absorption efficiency of a‐BTCG, we measured full UV–Vis–NIR absorption spectra (200–2500 nm) for a‐BTCG, u‐BTCG, Ti_3_O_5_ nanoparticles, and BN nanosheets (Figure 3b). Ti_3_O_5_ exhibits intense broadband absorption extending into the NIR region, whereas BN nanosheets show negligible intrinsic absorption but enhance internal photon scattering. As a result, the a‐BTCG evaporator displays significantly higher absorptance than the unaligned sample due to its vertically oriented channel architecture, which increases photon residence time through multi‐reflection and light trapping. Based on AM1.5G‐weighted integration, the broadband solar absorptivity of a‐BTCG is 89%, slightly lower than pure Ti_3_O_5_ (97%) because of the intrinsically low absorptance of BN. Nevertheless, the synergistic combination of broadband‐absorbing Ti_3_O_5_ and scattering‐enhancing aligned BN nanosheets enables highly efficient solar harvesting across the entire spectrum. Nevertheless, the introduction of BN nanosheets forms anisotropic multi‐level channels that induce multiple internal light scattering, significantly enhancing the effective utilization of incident photons. This optimized optical confinement improves photon delivery to the embedded Ti_3_O_5_ domains and facilitates efficient non‐radiative photothermal conversion, which is essential for achieving superior solar‐driven interfacial evaporation performance [41]. The a‐BTCG with vertically aligned channels exhibits rapid surface heating, with the temperature rising from 23.1  to 36.2 °C within 5 min, significantly faster than the disordered counterpart (Figure 3c,d). This enhanced thermal response is directly attributed to the directional alignment of BN, which facilitates efficient heat localization and in‐plane conduction.

**FIGURE 4:**
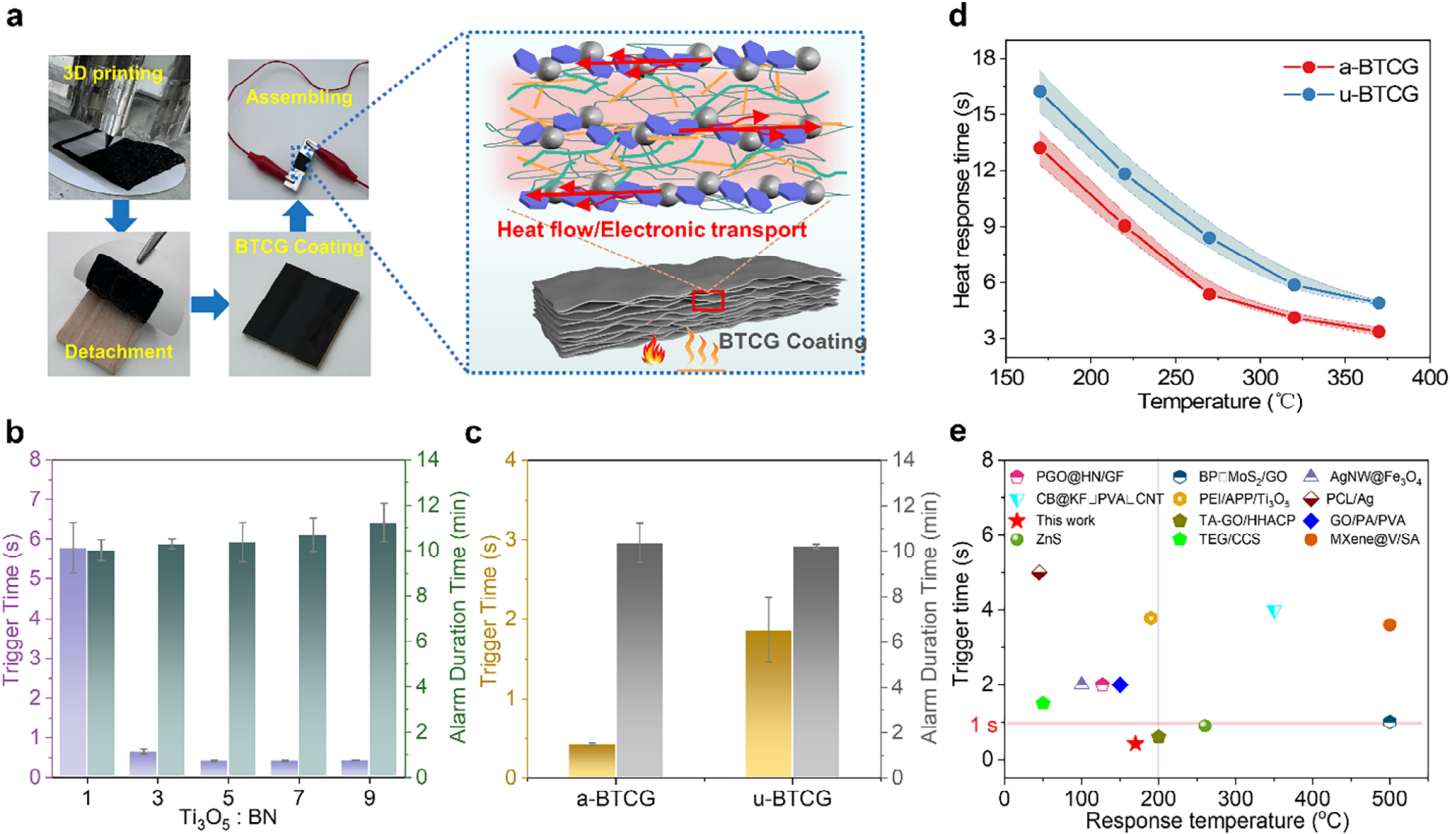
Thermal‐responsive capabilities of a‐BTCG. (a) Digital photographs illustrating the fabrication, coating, and assembly process of the thermal‐responsive devices, together with the schematic mechanism of heat‐induced electronic transport within the a‐BTCG coating. (b) Thermal‐triggered response behavior of Ti_3_O_5_/BN composites. (c) Enhanced and rapid thermal‐responsive behavior of a‐BTCG enabled by its anisotropic architecture. (d) Temperature evolution and heat‐trigger response times of a‐BTCG and u‐BTCG under different heating conditions, demonstrating fast activation and stable alarm duration. (e) Comparison of a‐BTCG with previously reported thermal‐responsive materials, highlighting its superior trigger speed and prolonged alarm duration.

To comprehensively evaluate the intrinsic thermal‐responsive behavior of a‐BTCG, which is a critical parameter of thermal conductivity, we evaluated its actuation performance under various thermal stimuli. A thermally responsive device was assembled using a‐BTCG (Figure 4a; Figure S15), with its structure showing no negative effect on mechanical integrity such as adhesion or hardness (Figure S16). Figure 4b shows the thermal‐triggered response behavior of Ti_3_O_5_/BN composites with different Ti_3_O_5_:BN ratios. Across the tested compositions, the trigger time remains within a narrow range, indicating that the incorporation level of Ti_3_O_5_ does not significantly affect the onset speed of thermal response. Upon thermal excitation, the aligned BN network enabled rapid in‐plane heat conduction, significantly accelerating the system's response. (Figure 4c) The a‐BTCG exhibits a minimum response time of 0.42 s, much faster than the 1.87 s observed in the disordered configuration. The response was also highly stable, with alarm signals consistently lasting 10–12 min. Temperature‐dependent actuation tests showed a sharp decrease in response time from 16.23  to 3.37 s within a 200°C increase. (Figure 4d), highlighting the effectiveness of anisotropic thermal pathways. This rapid response is attributed to the synergistic effect of BN‐induced directional heat transfer and the thermally activated electron transport within Ti_3_O_5_. The BN/Ti_3_O_5_ ratio is critical. Excessive BN impairs electron mobility due to its insulating nature, whereas insufficient BN compromises thermal pathway formation. Furthermore, the a‐BTCG demonstrates excellent cycling stability, maintaining consistent thermal responsiveness over multiple heating‐cooling cycles (Figure S17). When benchmarked against other thermally responsive materials, a‐BTCG shows a superior combination of fast actuation, structural robustness, and directional heat management (Figure 4e; Table S2), underscoring its potential for advanced thermal sensing and solar‐driven interfacial evaporation.

### 2.3 Solar‐Driven Interfacial Evaporation Performance Evaluation of a‐BTCG

**FIGURE 5:**
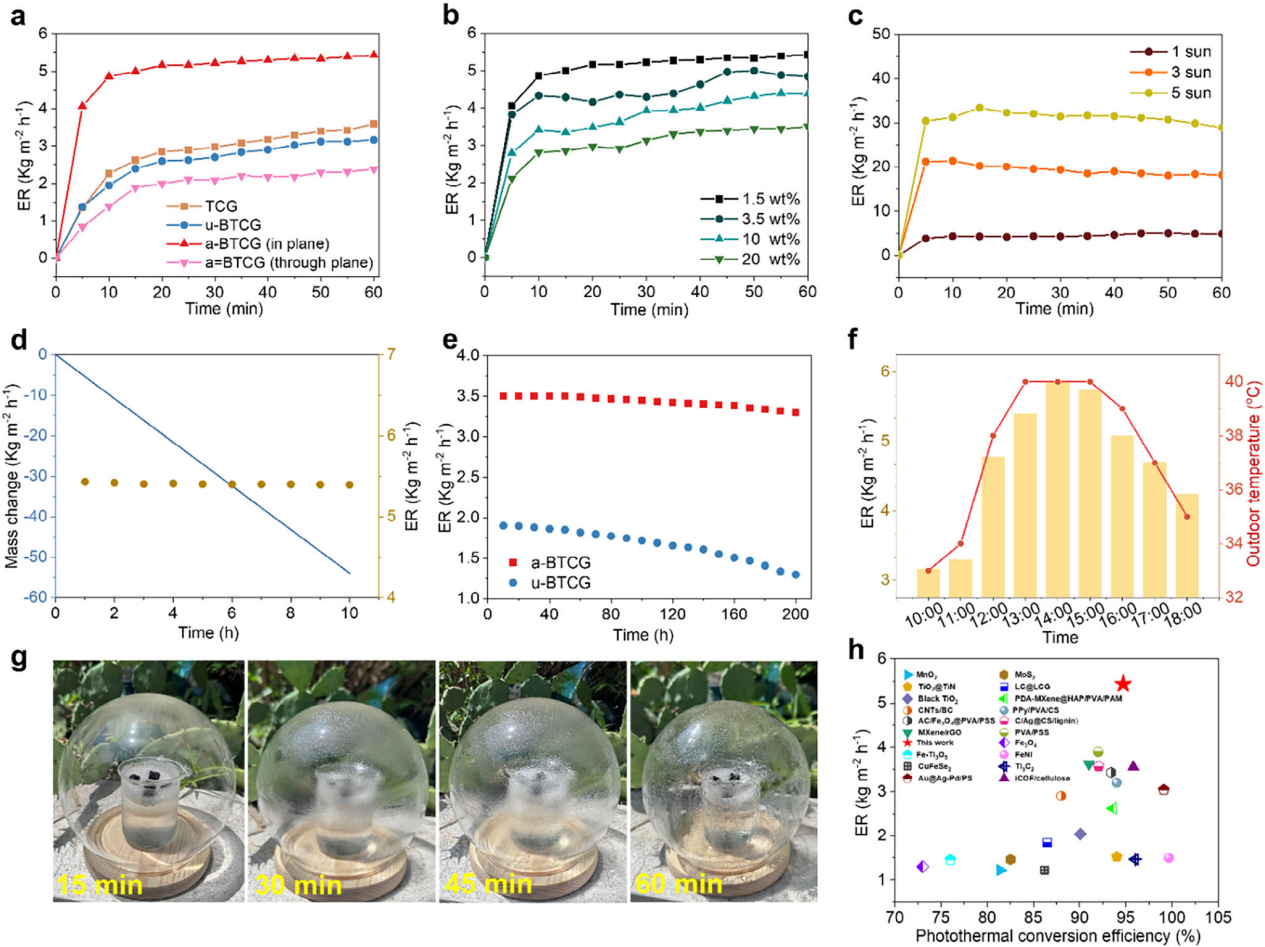
Solar‐Driven Interfacial Evaporation Performance Evaluation. (a) Evaporation rates of BTC, u‐BTCG, and a‐BTCG under 1 sun illumination, showing the performance enhancement induced by BN incorporation and directional alignment. (b) Evaporation performance of a‐BTCG in saline solutions of varying concentrations (0‐20 wt.% NaCl) under 1 sun illumination. (c) Evaporation rate of a‐BTCG under different solar intensities (1‐5 suns), demonstrating stable water supply and structural robustness at high flux. (d) Long‐term stability test of a‐BTCG in pure water under continuous 1 sun illumination. (e) Long‐term stability test of a‐BTCG in 20 wt.% NaCl, confirming excellent operational durability and salt resistance. (f) Outdoor evaporation performance of a‐BTCG across a full day, showing the correlation between evaporation rate and fluctuating ambient temperature/irradiance. (g) Rapid generation of condensed water droplets on the inner dome surface (15–60 min) under natural sunlight, visually demonstrating strong photothermal evaporation capability. (h) Comparison of the photothermal conversion efficiency and evaporation rate of a‐BTCG with state‐of‐the‐art solar evaporators reported in the literature.

To evaluate the practical photothermal performance of a‐BTCG, we systematically investigated its solar‐driven interfacial evaporation behavior under 1 sun illumination. (Figure S18) The a‐BTCG with vertically aligned channels exhibits rapid surface heating, with the temperature rising from 23.1  to 36.2 °C within 5 min, significantly faster than the disordered counterpart (Figure 3c,d). This enhanced thermal response is directly attributed to the directional alignment of BN, which facilitates efficient heat localization and in‐plane conduction. During the solar‐driven interfacial evaporation under 1 sun, the mass change of the a‐BTCG evaporator shows a linear relationship with illumination time, indicating stable and continuous evaporation performance. The a‐BTCG exhibits the largest mass change and the highest evaporation flux (5.43 kg m^−2^ h^−1^, Figure 5a). In contrast, the u‐BTCG shows a reduced evaporation rate due to its disordered channel geometry, while the TCG exhibits the lowest performance. These results clearly highlight the synergistic contributions of BN incorporation and directional channel alignment to efficient solar‐driven interfacial evaporation. To further evaluate the energy utilization efficiency, we conducted dark evaporation experiments to determine the effective enthalpy of water evaporation. We determined that the effective enthalpy of evaporation for a‐BTCG is 1003 J g^−1^, which is significantly lower than that of bulk water (∼2260 J g^−1^). This reduction of more than 55% and the energy efficiency of BCTG is 87.2%, indicating that the a‐BTCG structure effectively promotes interfacial water evaporation and reduces the overall energy requirement [42].

To further assess the practical desalination potential of a‐BTCG, we systematically investigated its performance at different salt concentrations (Figure 5b). Since seawater and brine conditions can vary widely in salinity, evaluating solar‐driven interfacial evaporation behavior across a range of NaCl concentrations (1.5, 3.5, and 10 wt.%) is essential to confirm the material's ion tolerance and sustained efficiency under more challenging conditions. Additionally, we explored the impact of varying light intensities (Figure 5c) to emulate real‐world solar conditions and assess the scalability of the system. By testing under 1 sun, 3 sun, and 5 sun illumination, we demonstrate that the a‐BTCG maintains stable performance with increasing solar input, further confirming its robustness and adaptability for practical solar‐driven interfacial evaporation applications.

To ensure the practical applicability of the a‐BTCG system under continuous operation, we further evaluated its long‐term photothermal evaporation stability. The hanging a‐BTCG can maintain the stable evaporation rate of ∼5.43 kg m^−2^ h^−1^ during 10 h irradiation at 1.5 wt.% NaCl solution (Figure 5d), without the solid‐salt crystallization. We first monitored the ion concentration and volume changes in the bulk salt solution during evaporation and found that the NaCl remained largely in the bulk solution. Furthermore, SEM and EDX analyses of the a‐BTCG after the stability tests revealed no NaCl accumulation at the top surface of the evaporator, demonstrating its excellent salt resistance. (Figure S19) To verify the structural advantages imparted by 3D printing, we performed a comprehensive mechanical analysis before and after long‐term testing. Uniaxial compression tests reveal that a‐BTCG exhibits a significantly higher compressive modulus of 0.8 MPa, compared with 0.36 MPa for the u‐BTCG, indicating that the ordered channel walls formed during printing serve as effective load‐bearing lamellae. The stress–strain profile of a‐BTCG further shows a stable deformation plateau and greater energy absorption, whereas the unaligned counterpart displays early pore collapse and brittle failure. Moreover, after long‐term desalination, the a‐BTCG retains 95% of its initial compressive modulus, demonstrating exceptional operational durability (Figure S20a). In addition, we measured the concentration of GA in the NaCl solution after the stability tests and found that there was almost no GA leakage, confirming its good chemical stability and effectively preventing potential secondary contamination. (Figure S20b)

To rigorously validate the long‐term desalination performance of a‐BTCG under harsh saline conditions, we conducted extended stability tests in 20 wt.% NaCl brine. Under 1 sun illumination, as shown in Figure 5e, the a‐BTCG evaporator maintained a highly stable flux of ∼3.3 kg m^−2^ h^−1^ over 200 hours of continuous operation (20 cycles × 10 h), corresponding to 94% retention of its initial performance (3.5 kg m^−2^ h^−1^). Post‐cycling SEM observations (Figure S21) show that the BTCG maintains its aligned porous architecture after 200 h operation in 20 wt.% NaCl, with no evidence of collapse, clogging, or structural deterioration. Although a small amount of NaCl crystallites can be seen on the channel surfaces, expected for samples directly removed from highly concentrated brine, these deposits are sparse, non‐continuous, and limited in size. They do not block the transport pathways nor alter the morphological integrity of the framework. This confirms that the a‐BTCG architecture effectively prevents salt accumulation within the channels and retains its structural stability under prolonged exposure to near‐saturated brine. XRD patterns (Figure S22) further verify that the characteristic diffraction peaks of the a‐BTCG framework are unchanged after cycling, demonstrating that the crystalline structure and phase composition remain stable under prolonged exposure to highly saline environments. These results confirm that the anisotropic architecture produced via 3D printing not only improves photothermal and water‐transport properties but also delivers higher mechanical robustness compared to u‐BTCG.

To evaluate outdoor solar‐driven interfacial evaporation performances, a‐BTCG fabric‐based evaporator was tested on a sunny day (Figure S23). Field experiments were held at Nanjing Forestry University in China on July 3, 2025, from 9:00 to 18:00. (Figure 5f,g) The environmental temperature rose from ∼33°C at 10:00 to ∼40°C at 13:00 and declined to ∼ 35°C at 18:00. Correspondingly, the evaporation rate was increased from 3.15 kg m^−2^ h^−1^ at 10:00 pm to 5.87 kg m^−2^ h^−1^ at noon and then decreased to 4.25 kg m^−2^ h^−1^ at 18:00. (Figure 5f). The higher evaporation flux observed around noon is fully consistent with the elevated ambient temperature, which naturally raises the interfacial temperature of the evaporator and enhances vapor‐generation kinetics. This behavior aligns with the well‐established dependence of evaporation rate on environmental temperature and humidity. Notably, the improved midday flux further indicates that the directionally organized composite structure of a‐BTCG effectively modulates the interfacial water layer and lowers the effective evaporation enthalpy, enabling robust and efficient evaporation under fluctuating outdoor thermal conditions. Figure 5 g shows that, within only 15–60 min under natural sunlight, a large amount of condensed water rapidly appears on the inner surface of the transparent dome. This fast condensation visually demonstrates the strong photothermal evaporation capability of the a‐BTCG evaporator, even in a relatively enclosed environment. Compared to conventional porous materials, this synergistically engineered structure exhibits superior photothermal and water‐handling performance, as evidenced by benchmark comparisons. (Figure 5 h; Tables S1 and S2) The performance of a‐BTCG in desalination is also compared with other photothermal materials, showcasing its exceptional comprehensive performance. These significant advantages are attributed to the directional structural design that controls the transport paths of phonons, photons and water, reducing energy losses to achieve excellent thermal management performance.

### 2.4 Mechanistic Insights: Anisotropic Structure Enhances Thermal Management and Water Transport

**FIGURE 6:**
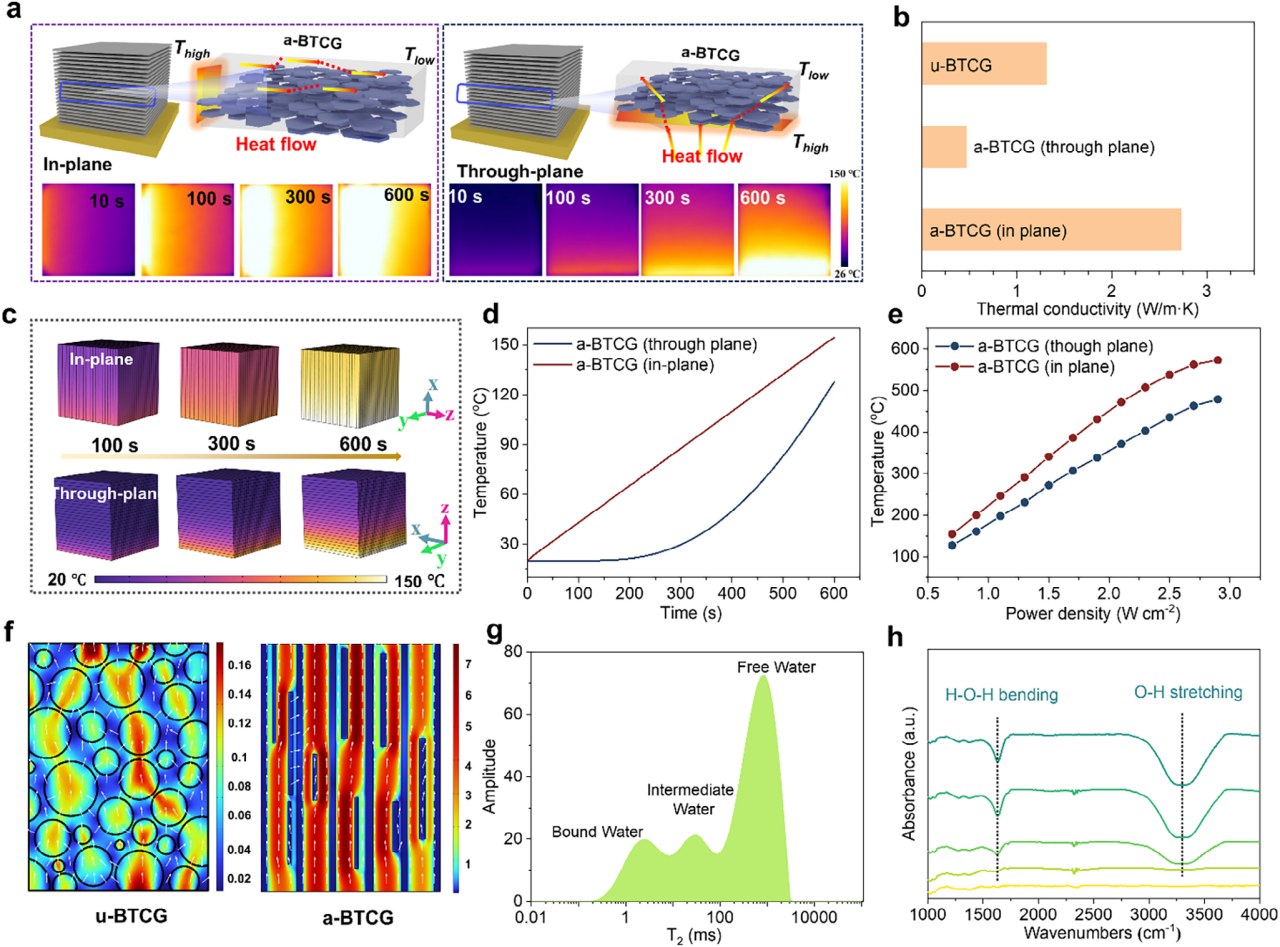
Directional Transport Mechanisms Enabled by Anisotropic Architecture. (a) Schematic illustration of anisotropic heat flow in a‐BTCG resulting from BN nanosheet alignment. (b) Measured thermal conductivity of u‐BTCG and a‐BTCG along the in‐plane and through‐plane directions, confirming strong directional contrast. (c) COMSOL‐simulated transient thermal‐transfer profiles comparing in‐plane and through‐plane heat propagation (qualitative visualization). (d,e) Corresponding simulated temperature‐time curves and temperature‐power‐density relationships, highlighting faster in‐plane thermal equilibration. (f) Simulated distribution of confined water layers within u‐BTCG (left) and a‐BTCG (right), showing enhanced continuity and reduced tortuosity in aligned architectures. (g) Low Field NMR spectra of wet a‐BTCG, revealing bound, intermediate, and free water populations. (h) In situ FTIR curves of a‐BTCG at different water decreasing.

The anisotropic architecture of a‐BTCG, arising from the directional alignment of BN nanosheets, plays a pivotal role in enhancing both thermal management and water transport capabilities. Experimental comparison of in‐plane and through‐plane thermal conduction in a‐BTCG reveals a marked dependence on BN orientation. When heat propagates along the alignment direction, the thermal conductivity reaches 2.73 W m^−1^ K^−1^, significantly higher than the through‐plane value of 0.47 W m^−1^ K^−1^ (Figure 6a,b). In contrast, u‐BTCG exhibits a lower, isotropic conductivity of 1.31 W m^−1^ K^−1^. This pronounced anisotropy stems from shear‐induced BN alignment, which forms continuous thermal pathways that facilitate efficient phonon transport.

Moreover, the nanoscale quantum confinement effect within the aligned BN layers further enhances phonon directionality by limiting scattering events, thereby boosting overall thermal conduction. Also, due to their highly oriented ‘brick‐mortar’ layer structure, Ti_3_O_5_ particles can also act as ‘thermal bridges’ to enhance the thermal conduction pathways at the CS/BN and BN/BN interfaces, further reducing interfacial thermal resistance and reinforcing in‐plane heat transfer. To delve deeper into the influence of the anisotropic thermal conductivity of BN structures on the heat transfer process, finite element analysis of transient heat conduction was performed (Table S3; Figures S24 and S25). The transient thermal transfer process is depicted in Figure 6c. It is evident that as time progresses from 0 to 600 s, the BN through‐plane exhibits a quicker heat transfer process. From the detailed temperature profile shown in Figure 6d, the final temperature of the BN through‐plane is 27°C higher than that of the BN in‐plane. As the power density increases, the temperature difference between the BN through‐plane and in‐plane widens (Figure 6e). To highlight the unusually fast thermal response of the a‐BTCG evaporator, we quantitatively compared its heating kinetics and anisotropic thermal conductivity (Table S4). This comparison confirms that a‐BTCG possesses one of the fastest temperature rise rates among architected or printed evaporators. This accelerated heating behavior originates from the vertically aligned BN nanosheets, which create continuous, low‐resistance in‐plane phonon pathways. The aligned BN structure also suppresses out‐of‐plane heat dissipation, enabling efficient heat localization at the evaporation interface. These results underscore the critical role of structural design in governing thermal energy flow.

Beyond thermal management, the vertically aligned channel structure also facilitates directional water transport. As demonstrated previously, contact angle measurements confirmed the inherent hydrophilicity of the CS matrix (Figure 2i), essential for capillary‐driven water transport. The CS matrix forms interconnected capillary pathways, which would structure the water layers with its intrinsic hydrophilic. Figure 6f shows the theoretical simulation of water distribution and transport within the material's porous network. Compared to the u‐BTCG (water flux: 2.97 × 10^−4^ µm^3^ s^−1^), the aligned structure exhibits over 35‐fold improvement in water flux (1.13 × 10^−2^ µm^3^ s^−1^), attributed to lower mass transfer resistance and optimized pore geometry. Notably, the green areas adjacent to the pore walls correspond to bound water, which exhibits restricted mobility due to strong interactions with the material interface. Despite its limited diffusivity, this bound water layer provides well‐defined transport channels that promote directional water migration across the structure. A clear contrast between the disordered and ordered architectures highlights the enhanced connectivity and anisotropic transport pathways enabled by the ordered arrangement. Low‐field nuclear magnetic resonance (LF‐NMR) was further used to study the distribution of water within the a‐BTCG. The results indicate three distinct states, bound water, intermediate water, and free water, characterized by their T_2_ relaxation times. (Figure 6g) The excellent agreement between the experimental LF‐NMR data and the theoretical model corroborates the presence of multiple water environments and validates the proposed transport mechanisms [43]. In situ FTIR measurements further confirm effective water transport and vaporization, as indicated by the progressive attenuation of hydroxyl absorption bands during evaporation (Figure 6h). Overall, these results demonstrate that the engineered anisotropic structure of a‐BTCG not only enables high thermal conductivity and rapid heat dissipation but also supports efficient directional water flow and light utilization, key features for next‐generation solar‐driven evaporation systems.

## 3 Conclusion

In this work, we established a bio‐inspired dual‐anisotropy design strategy for solar‐driven interfacial evaporation by integrating geometric channel alignment with materials‐level orientational ordering. Using carefully engineered 3D‐printing ink, we enabled shear‐induced alignment of BN nanosheets while uniformly embedding photothermal Ti_3_O_5_ nanoparticles and a hydrophilic chitosan network within a hierarchically porous scaffold. This programmable architecture, conceptually inspired by the vertically oriented vascular tissues of natural wood, creates a coupled photon‐phonon‐water transport pathway that cannot be achieved through geometric anisotropy alone. The a‐BTCG framework enhances full‐spectrum light harvesting through multireflection channels, establishes continuous in‐plane phonon pathways for rapid and localized thermal regulation, and reorganizes interfacial water into a structured hydration layer that lowers effective evaporation enthalpy. These cooperative interactions overcome fundamental transport bottlenecks in conventional evaporators, enabling an ultrahigh evaporation rate of 5.43 kg m^−2^ h^−1^ under 1 sun, a directional thermal conductivity of 2.73 W m^−1^ K^−1^, rapid thermal‐response times down to 0.42 s, and excellent stability in 20 wt.% saline for over 200 h. Our results highlight the critical importance of materials‐level anisotropy in advancing solar‐thermal systems and demonstrate the unique capability of 3D printing to construct tailored, multifunctional architectures with precise control over transport phenomena. This anisotropy‐guided design framework provides a generalizable route for engineering next‐generation photothermal, thermal‐management, and water‐processing materials, with promising implications for desalination, environmental remediation, and intelligent sensing technologies.

## 4 Methods

### 4.1 Chemicals and Reagents

Ti_3_O_5_, black powder with metallic luster (O element content = 62.3 at%∼64.3 at%, ρ = 4.29 g cm^−3^, orthorhombic structure), chitosan (CS, deacetylation degree: 80.0‐95.0%), boron nitride nanosheets (BN, 99.9% metals basis, 1–2 µm, MW 24.8) and Glutaraldehyde (GA, 20 wt.%) was bought from Aladdin Reagent Co., Ltd. (Shanghai, China). BN was pretreated via a wet ball milling method assisted with urea (NH_2_)_2_CO modification. Glacial acetic acid (HAc, 99.5%) was kindly supplied by Sinopharm Chemical Reagent Co., Ltd. All reagents used in this experiment were used directly without further purification. Wood (*fir spp*.) bought from Guangxi Province was further sawn into small pieces with the desired dimensions.

### 4.2 Synthesis of BN Nanosheets

BN nanosheets were prepared by the urea‐assisted ball milling method. First, commercial BN micro‐powder with a size range of 1–2 µm and urea were mixed in a stainless‐steel milling container with a weight ratio of 1:50 (BN/urea). Using a planetary ball mill (QM‐QX 2), the mixture of BN and urea was ball‐milled at a rotation speed of 400 rpm for 20 h. The excess amount of urea induces functionalization and also protects BN from mechanical damage. After the ball milling process, the mixture was dialyzed with a cellulose semipermeable membrane (3500 kDa, Whatman) in deionized water for 3 days to remove excess urea. The obtained BN aqueous dispersion was centrifuged at high speed (4000 rpm, 10 min) to collect sediment BN and remove water. After that, the collected BN would be redispersed in deionized water by simple ultrasonication (900 W) for 1 h. Then the redispersed solution was centrifuged again in moderate conditions (3000 rpm, 30 min) to remove bulk BN particles and collect high aspect ratio BN nanosheets. Finally, the collected supernatant with a high aspect ratio of BN dispersion was obtained (Figures S1 and S2).

### 4.3 Fabrication of 3D Printing Ink

A 2 wt.% CS solution was prepared with acetic acid solution (pH = 4). Ti_3_O_5_ powder was dispersed in 10 mL of CS solution, and 10 mL of BN solution (2.5 wt.%) was mixed. The mass ratio of BN to Ti_3_O_5_ was 1.25:1, 1.25:3, 1.25:5, 1.25:7, and 1.25:9 and denoted as Ti_3_O_5_/BN 1, Ti_3_O_5_/BN 3, Ti_3_O_5_/BN 5, Ti_3_O_5_/BN 7, Ti_3_O_5_/BN 9, respectively. The solution was stirred for 1 h to obtain a homogeneous dispersion. To obtain a gel ink with rheology satisfying 3D printing, 0.125 mL of GA solution with a concentration of 20 wt.% was added to CS/BN/Ti_3_O_5_. It was stirred for 1 h until CS crosslinked with GA to produce a gel. The prepared inks were homogenized by centrifugation under vacuum for 18 min to eliminate foam and stored at 5°C. The mass ratio of BN to Ti_3_O_5_ was 1.25:5 (Ti_3_O_5_/BN 5) is chosen to further study its morphology and structure due to the best printability as well as thermal performance (Figures S5–S14).

### 4.4 3D Printing with Ink

The 3D printing process was carried out using a Dr. INVIVO 4D bioprinter (ROKIT Healthcare, INC, South Korea) equipped with a pneumatic dispenser. The print ink was transferred into a 20 mL syringe (needles with an inner diameter of 0.4 mm) and installed into the 3D printer. Cinema 4D and NewCreator K were used to construct the 3D model as well as to set the printing parameters. The infill pattern was a grid with 90% infill density, 0.4 mm per layer height, and 4 mm s^−1^ printing speed. Then store the finished print at 5°C and frozen at −18°C and then freeze‐dried for 48 h to obtain the self‐standing 3D architectures.

## Author Contributions

S.S., S. Z., Y.Y., and M.P. conceived the idea and designed the experiments. Y.Y. and M.P. supervised the project. S.S. constructed/optimized the materials. Z.H and C.Z carried out theoretical calculations and analysis. S.Y., C.M., and D.T. provided the resources. S.S. and D.J. reviewed the data. S.S. and D.J. wrote the manuscript. All authors discussed the results and were engaged in revising and completing the final version of the manuscript.

## Conflicts of Interest

The authors declare no conflicts of interest.

## Supporting information




**Supporting File**: advs73923‐sup‐0001‐SuppMat.docx.

## Data Availability

The data that support the findings of this study are available from the corresponding author upon reasonable request.
