# Supplementary material for: 3D‐Printed Architected Anisotropic Channels for Ultrafast Solar‐Driven Interfacial Evaporation via Localized Thermal Management and Water Layer Structuring
Source: Adv Sci (Weinh). 2026 Jan 28;13(18):e20694. doi: 10.1002/advs.202520694 (PMC13042679; doi:10.1002/advs.202520694)
Supplement: Supplementary file 1 — Supporting File: advs73923‐sup‐0001‐SuppMat.docx. [file ADVS-13-e20694-s001.docx]

Supporting Information

**3D-Printed Architected Anisotropic Channels for Ultrafast Solar-Driven Interfacial Evaporation via Localized Thermal Management and Water Layer Structuring**

*Sijia Sun, Dong Jiang*, Hengsong Zheng, Shuai Zhang, Ziyuan Cheng, Changtong Mei, Dan Tian, Shilong Yang, Yusuke Yamauchi*, Mingzhu Pan**

Supplementary

**Figure** S1-S25

**Table** S1-S4

**Charaterizations:**

All physical characterizations were conducted using calibrated instruments with defined measurement accuracy and operating ranges. Transmission electron microscope (TEM, JEM-1400, Rigaku, Japan) are used to image formation for BN. Scanning electron microscopy (FE-SEM, QUANTA 200, FEI, USA) was conducted to investigate the microscopic structure of treated specimen. X-ray diffraction (XRD, X' TRAX, ARL) was performed to determine the orientational arrangement of BN. As a radiation source, CuKα has a wavelength of λ=1.5406Å, a scanning range of 2θ from 10° to 50° at a speed was 5°/min. X-ray photoelectron spectroscopy (XPS) analysis was carried out using an electron spectrometer and an Al Kα line excitation source, with the C 1s at 285.0 eV as a reference. Rheological properties of the printing inks were measured using a ThermoFisher MARS60 rotational rheometer, equipped with a 25-mm parallel-plate geometry, torque resolution of 0.1 µN·m, and shear-rate range of 0.01–1000 s⁻¹. Solar absorption spectra (200–2500 nm) were recorded using a PerkinElmer Lambda 1050 UV–Vis–NIR spectrophotometer with a dual-detector system (PMT/InGaAs) and a spectral resolution of 1 nm. Thermal images were captured using a FLIR E95 infrared camera with a thermal sensitivity of <0.05 °C, emissivity calibrated to 0.95. Thermal conductivity was measured using a DRPL-5 transient plane-source conductivity meter, with a measurement accuracy of ±3% and an operating range of 0.01–5 W·m⁻¹·K⁻¹. LF-NMR measurements were performed on a PQ001 benchtop NMR analyzer (12.5 MHz, CPMG sequence), with a relaxation-time detection limit of 0.1 ms. FTIR spectra were acquired on a Bruker VERTEX 80 spectrometer with a spectral resolution of 0.5 cm⁻¹. SAXS patterns were obtained using an Anton Paar SAXSess MC2 system with a q-range of 0.01–2.5 Å^-1^. All indoor solar-driven interfacial evaporation measurements were conducted using a Newport 94023A solar simulator equipped with an AM 1.5G filter and calibrated to 1000 ± 5 W·m^-2^ using a NIST-traceable Newport 91150V pyranometer. For outdoor experiments, irradiance was monitored in real time using a Delta-T SPN1 sunshine pyranometer with a measurement uncertainty of ±3%. Simulations of anisotropic thermal transport were performed using COMSOL Multiphysics, with thermal boundary conditions and material parameters taken from experimental measurements.”


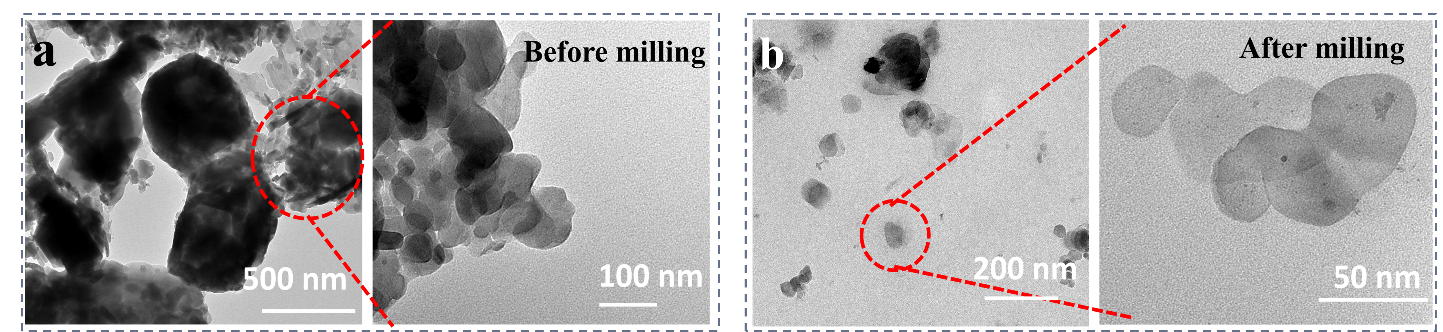


**Figure S1** TEM images of BN before (a) and after milling (b).


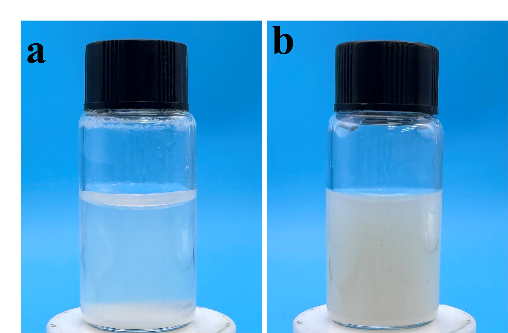


**Figure S2** Suspensions of BN before and after milling was left to stand for one week.


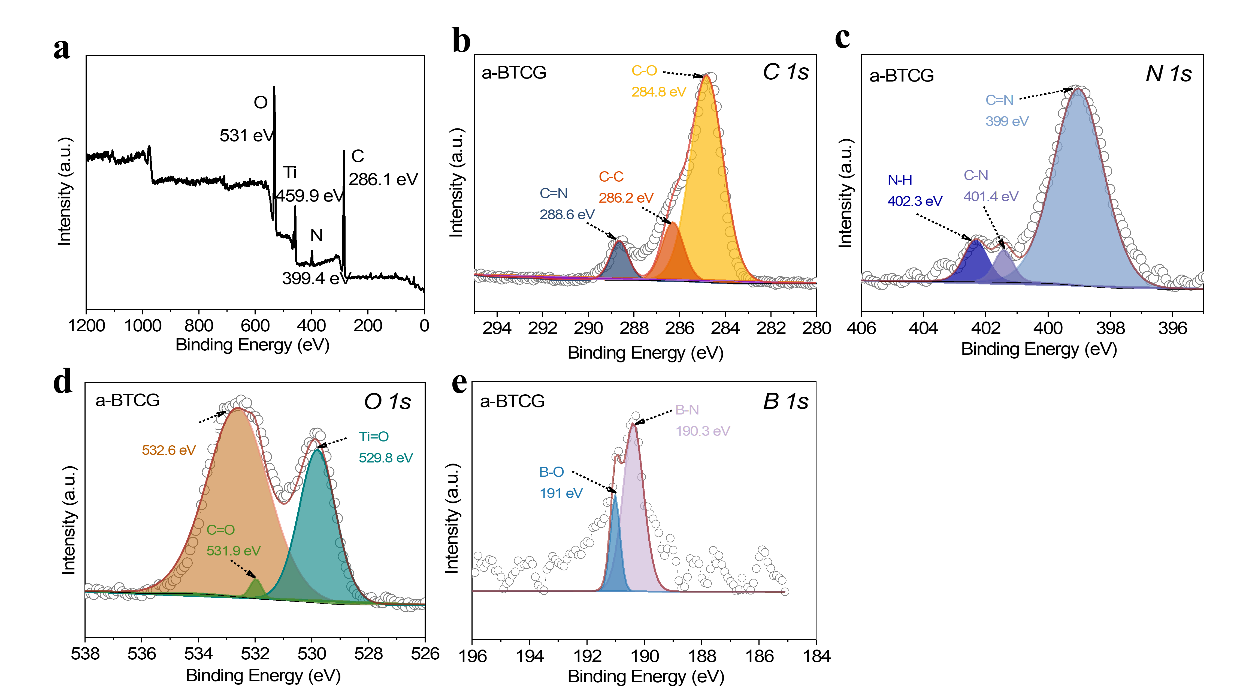


**Figure S3** XPS spectra of a-BTCG: incluing high resolution of C1s, N1s, O1s and B1s.


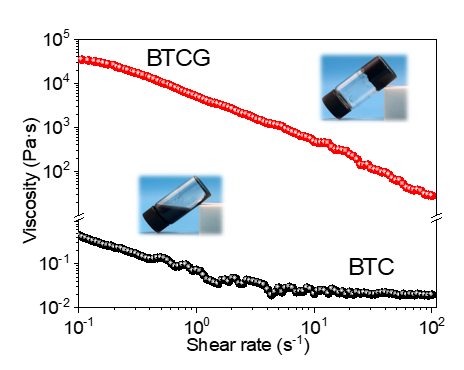


**Figure S4** The viscosity-shear rate of BTC and a-BTCG at room temperature.


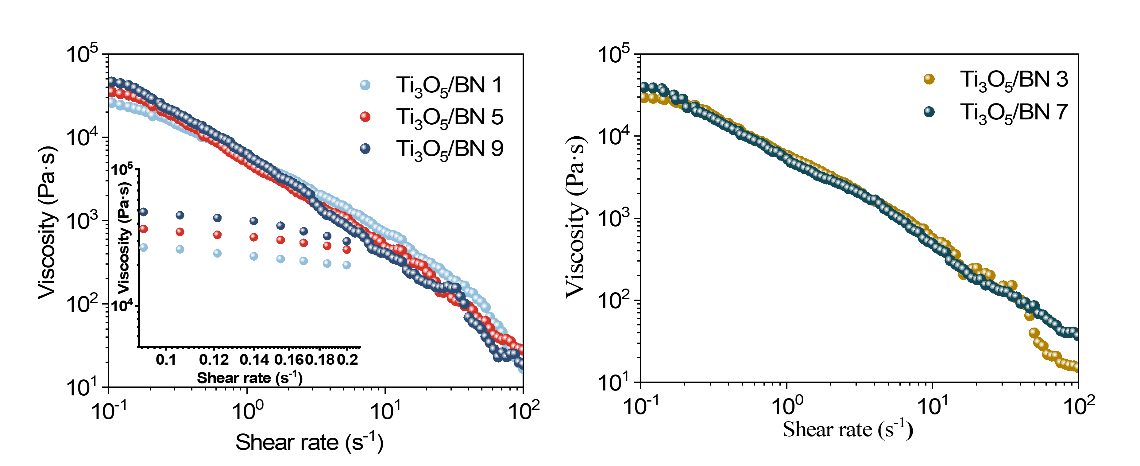


**Figure S5** The viscosity as a function of shear rate.

To optimize 3D printing inks, it is essential to finely tune the rheological properties, which are influenced by the choice of fillers, binders, solid content, and solvents. In direct ink writing for 3D printing, the rheological properties of the ink critically affect its printability, including extrudability, filament formation, shape fidelity, and geometrical accuracy. As illustrated in **Figure S5**, the viscosity of a-BTCG gel inks modestly increases with higher Ti_3_O_5_ loadings, displaying pronounced shear-thinning behavior under high shear stress which facilitates flow through the nozzle.


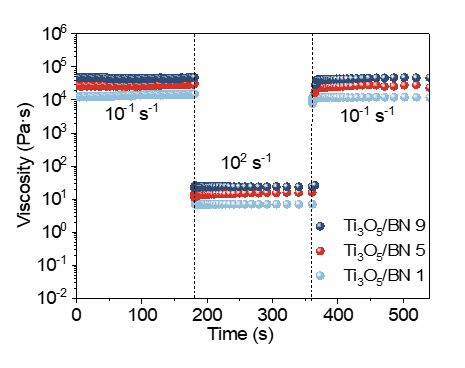


**Figure S6** Viscosity evolution over time for alternating low and high shear rates.

The thixotropic nature of the ink was assessed by alternating low and high shear rates, as depicted in **Figure S6**. Initially, a very low shear rate of 10^-1^ s^-1^ was applied to mimic pre-extrusion conditions. After continuous mixing at this rate for 180 s, the shear rate was elevated to 10^2^ s^-1^ to simulate the ink's extrusion through a narrow nozzle. The viscosity sharply decreased under higher shear and swiftly recovered upon reverting to the initial low shear rate, indicating the reformation of the BTCG gel network.


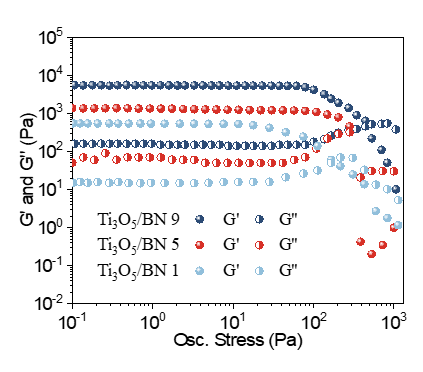


**Figure S7** G′ and G″ as a function of oscillatory stress.

The viscoelastic properties were further explored through dynamic rheological measurements, evaluating the ink’s response to oscillatory stress and angular frequency. Notably, the ink exhibits predominantly solid-like behavior below the crossover point (G’>G’’), transitioning to liquid-like behavior as stress exceeds the yield stress at the crossover points of G’=G’’. However, the networked structure collapses, leading to a solid-to-liquid transition occurs (**Figure S7**). The yield stress of inks demonstrated a marked increase from 140 Pa to 600 Pa, following the power law relationship:

τ_y=kΦ^p (1)

Here, τy is the yield stress, Φ denotes the volume fraction of particles, k is a constant determined by the particle interactions, size, and quantity, and p is an exponent reflecting microstructure of the particle network. This highlights the critical influence of inorganic filler content on the printability of the inks. As Ti_3_O_5_ loading increases, both the storage modulus (G') in the plateau region, where G' remains relatively constant under stress, and the yield stress increases.


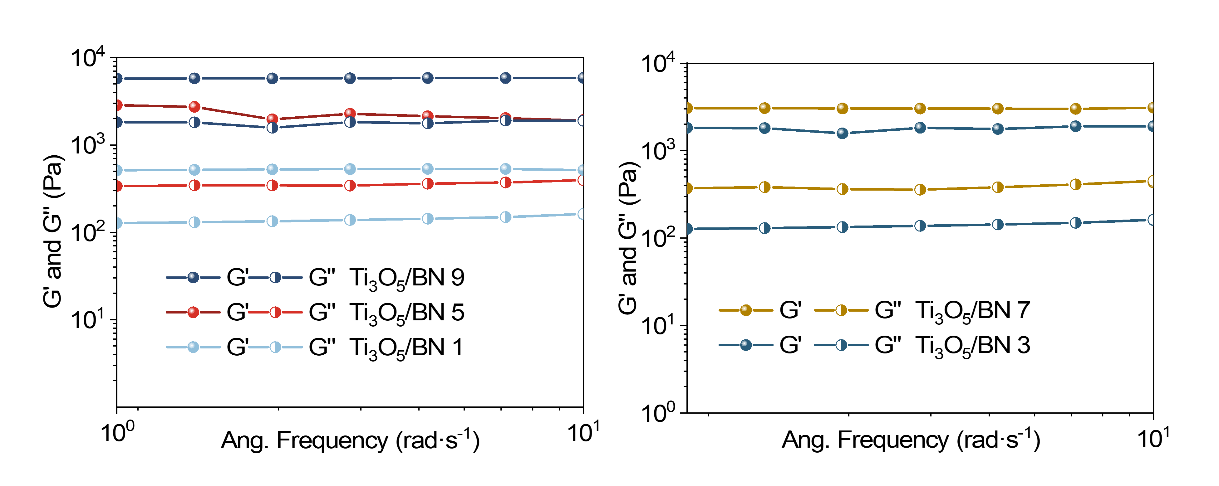


**Figure S8** Viscoelastic properties: storage modulus (G′) and loss modulus (G″).

Additionally, angular frequency sweeping measurements reveal that the inks maintain a high storage modulus (G') and complex modulus, demonstrating independence from angular frequency.


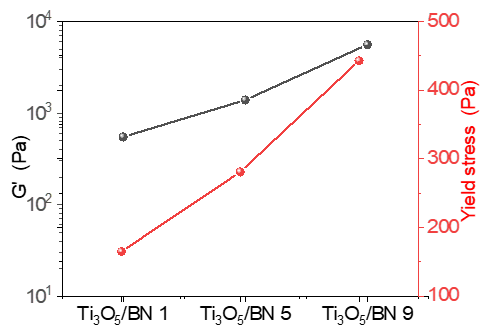


**Figure S9** G′ at plateau regions and yield stress obtained at the crossover point between G′ and G″.


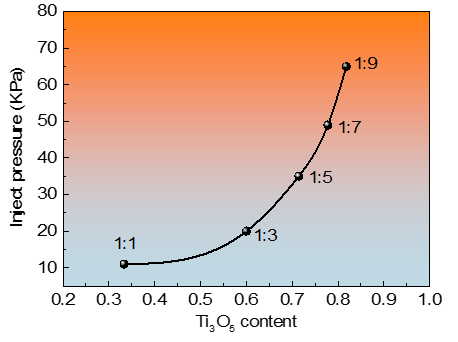


**Figure S10** Extrusion pressure of ink under different Ti_3_O_5_ content.

Inject pressure tests further indicated that the pressure required for ink extrusion increased with increasing Ti_3_O_5_ content.


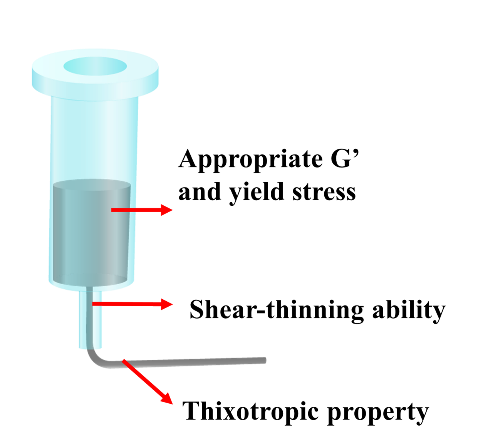


**Figure S11** Schematic diagram of the rheological properties required for 3D printable gel inks.


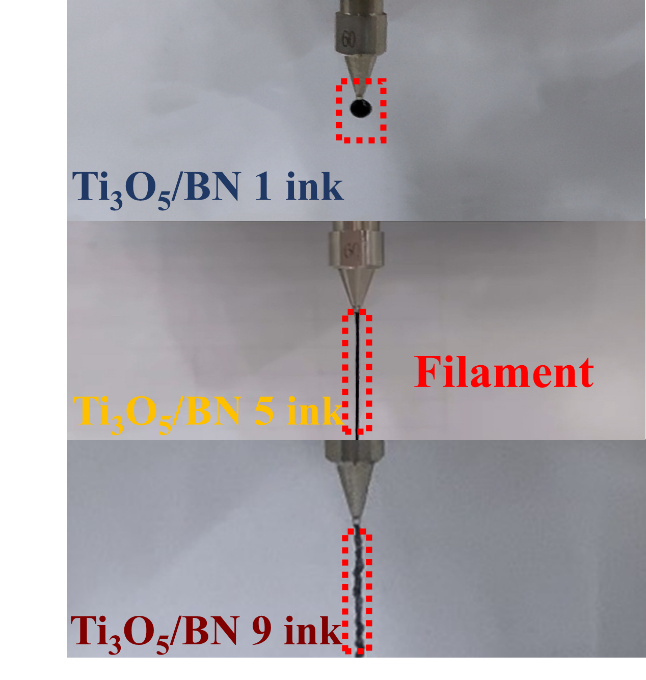


**Figure S12** Digital appearance of Ti_3_O_5_/BN inks upon extruding.

The as-prepared gel inks were loaded into a syringe equipped with a 0.4 mm diameter needle to evaluate their 3D printability. The Ti_3_O_5_/BN 5 ink produced a smooth, continuous filament due to its uniform composition and optimized rheological properties. However, the extruded filaments of Ti_3_O_5_/BN 1 and 9 were discontinuous, and clogged needles could not be extruded due to low and excessive solid content of Ti_3_O_5_, respectively. A low Ti_3_O_5_ content reduces the degree of gelation of the ink, which does not maintain shape stability after extrusion. On the contrary, the smooth extrusion of filaments is hindered by the thick and uneven particle size of excess Ti_3_O_5_. The yield stress of inks demonstrated a marked increase from 140 Pa to 600 Pa as shown in **Figure 3c**, following the power law relationship:

$\tau_{y}=k\Phi^{p}$ (2)

Here, $\tau$_y_ is the yield stress, *Φ* denotes the volume fraction of particles, *k* is a constant determined by the particle interactions, size, and quantity, and *p* is an exponent reflecting microstructure of the particle network. This highlights the critical influence of inorganic filler content on the printability of the inks. Models predict that for an isolated anisotropic particle in a Newtonian fluid, the alignment time adheres to a defined relationship:

t=(β(Φ))/(γ̇) (3)

This indicates that in concentrated (Φ) inks subjected to high shear rates (γ̇) particles align faster. Therefore, the ink concentration affects the orientation performance.


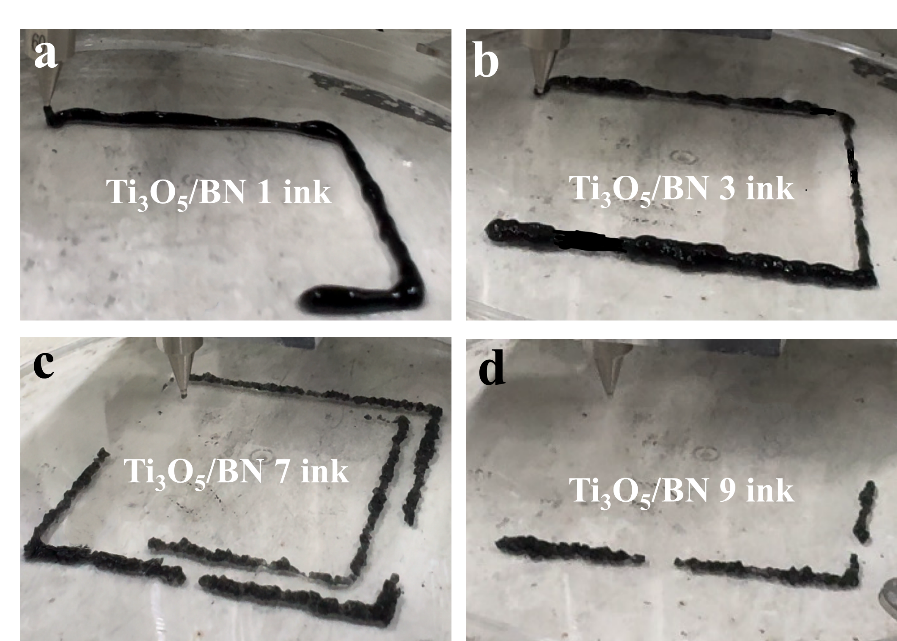


**Figure S13** Printing diagram of different Ti_3_O_5_/BN ink ratios.


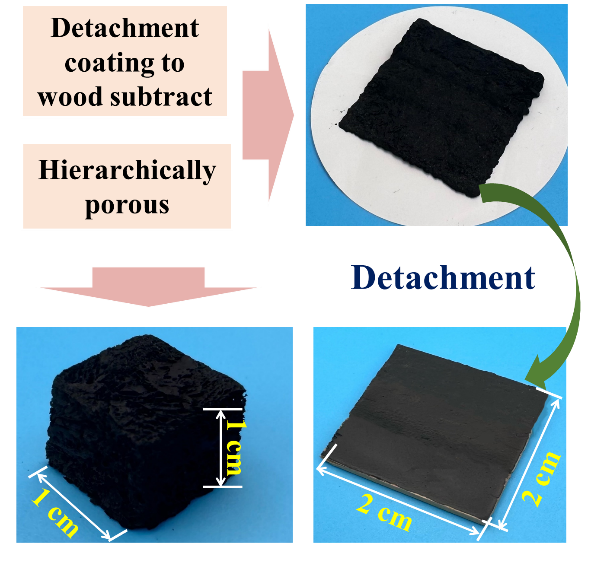


**Figure S14** Different 3D printed architectures using the Ti_3_O_5_/BN 5 ink.

Owing to the excellent 3D printability of Ti_3_O_5_/BN 5 ink, various 3D architectures were successfully fabricated. These structures maintain precise geometry and remain stable without any signs of deformation or collapse.


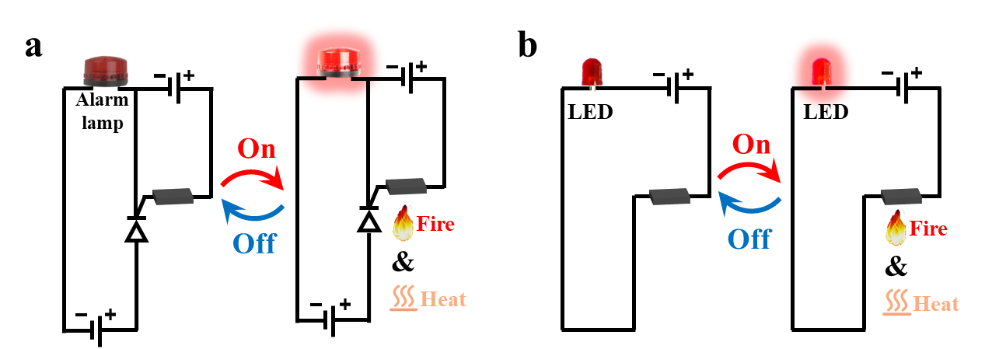


**Figure S15** Trigger warning circuit system.


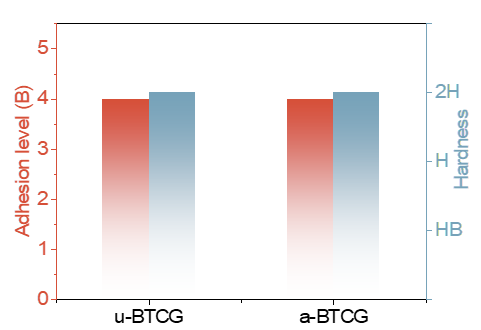


**Figure S16** Mechanical properties of a-BCTG and u-BTCG.


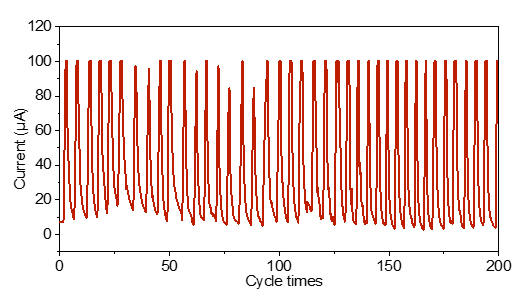


**Figure S17** Temperature current cycle curves of a-BTCG.


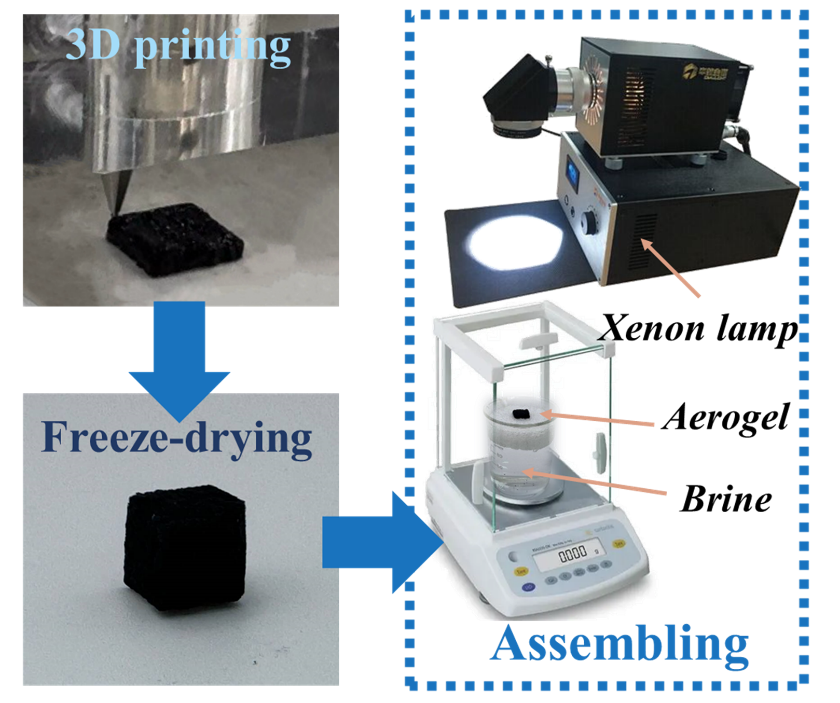


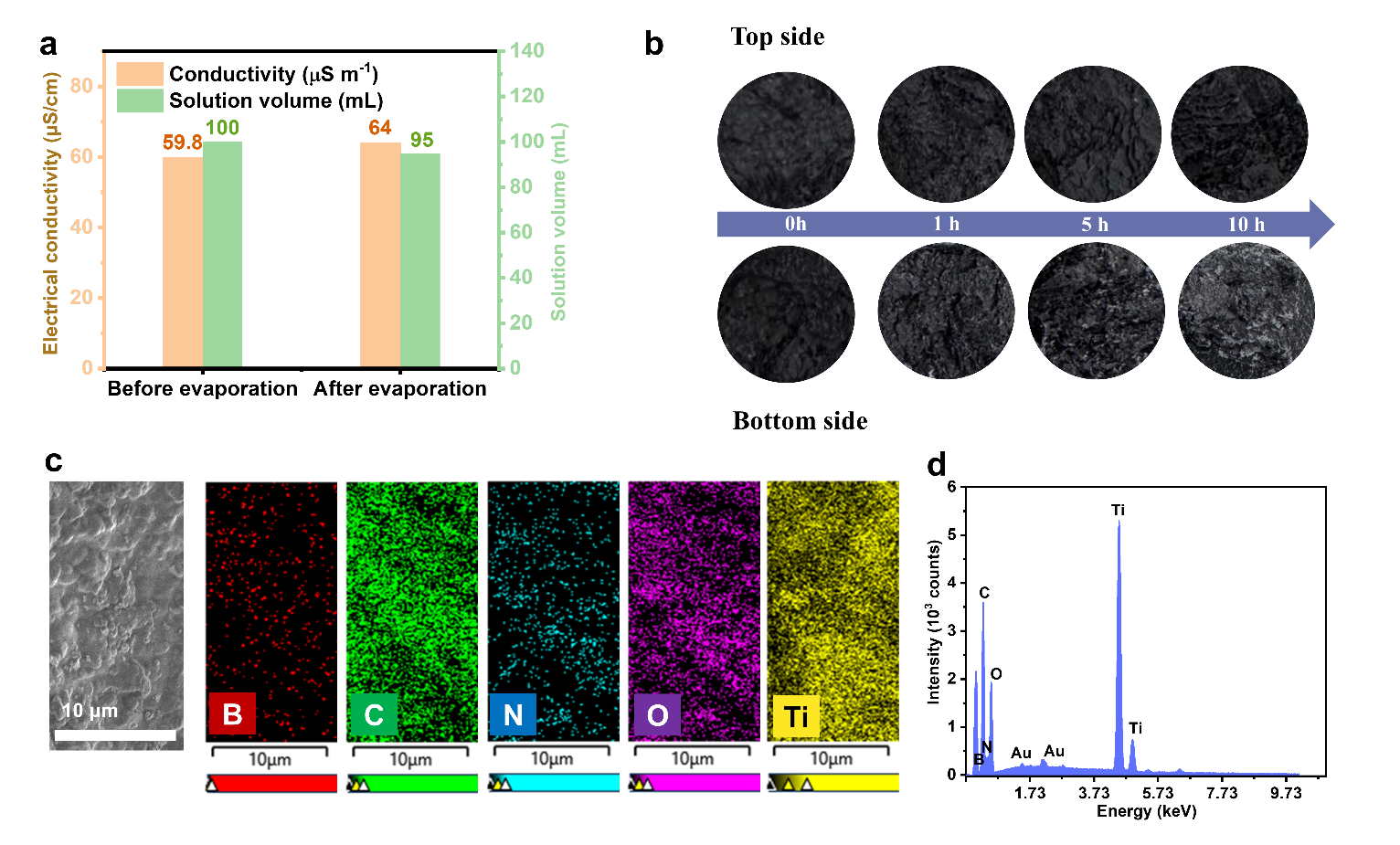
**Figure S18** Digital images of the fabrication process of solar-driven interfacial evaporator

**Figure S19** Salt resistance property of a-BTCG during the solar -driven interfacial evaporation. a) Concentration of NaCl evaluation of the bulk solution. b) Time-dependent images of the BTCG evaporator. The bottom side (immersed in NaCl solution) shows slight salt crystallization after 5 h, whereas the top side remains free of salt deposits, indicating effective salt resistance. c) SEM image and corresponding EDX elemental mapping of B, C, N, O, and Ti elements, indicating uniform distribution throughout the a-BTCG structure after long-term solar-driven evaporation. d) EDX spectrum further confirming the presence of B, C, N, O, and Ti elements, with no signals of NaCl deposits, highlighting the excellent anti-salt-fouling property.


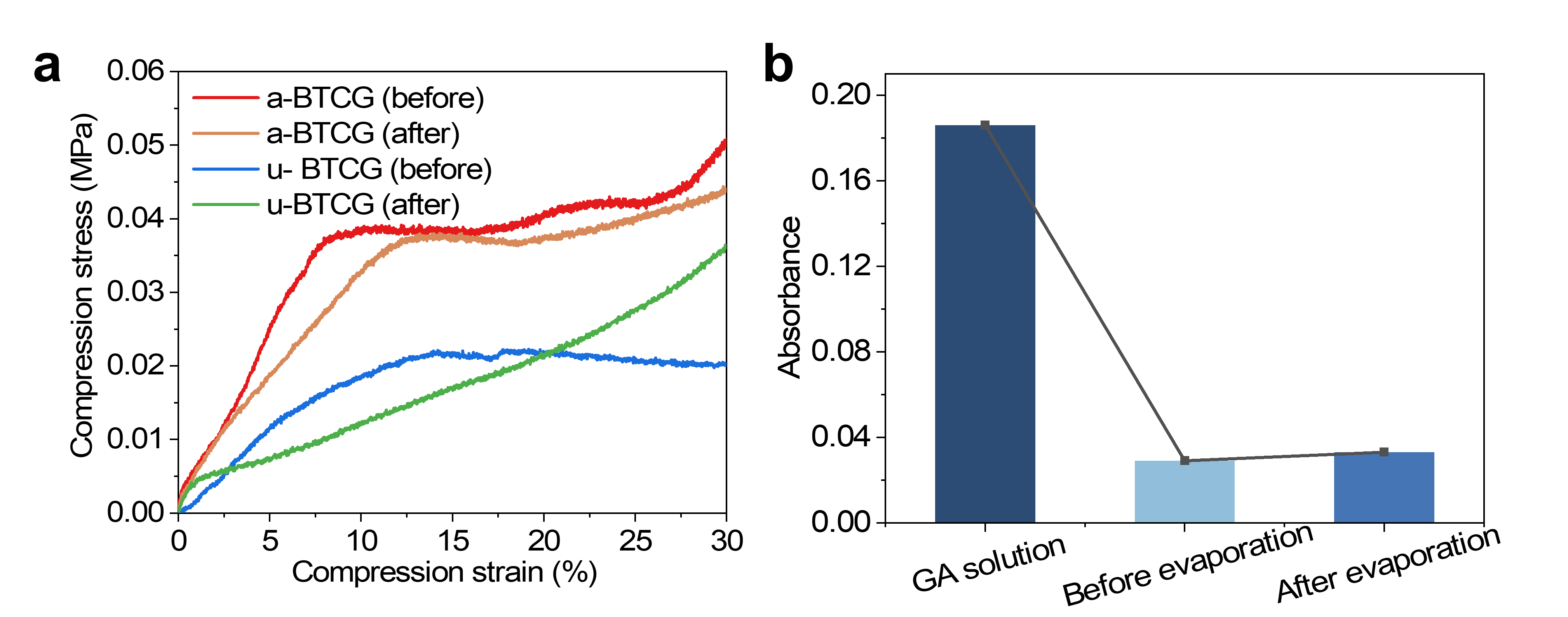


**Figure S20** Structural stability evaluation of the a-BTCG after long-term solar-driven interfacial evaporation test. a) Mechanical properties of the a-BTCG and u-BTCG before and after long-term solar-driven evaporation. b) The concentration of GA in the bulk NaCl solution displays a slight difference after a long-term solar-driven interfacial evaporation test, suggesting a stable chemical structure of a-BTCG.


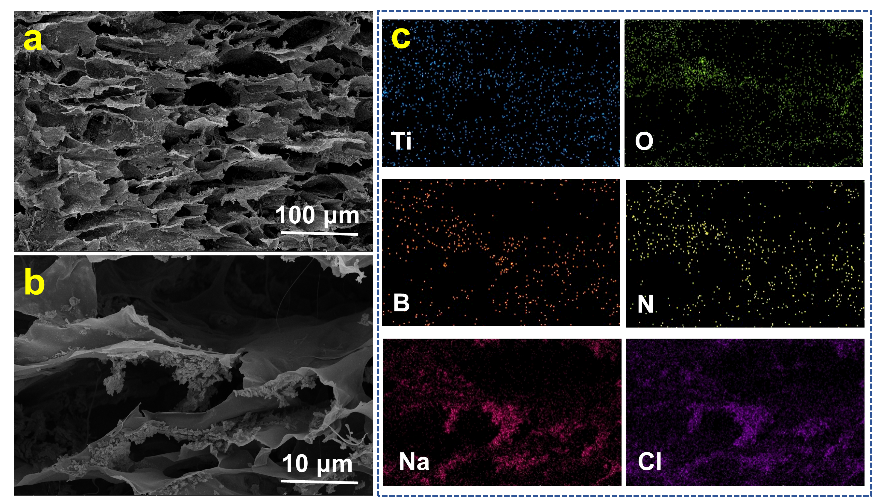


**Figures S21** The SEM and EDS image of a-BTCG from the side view after 200 h solar-driven interfacial evaporation.

**Figures S22** XRD patterns of directional structure analysis after 200 h solar-driven interfacial evaporation.


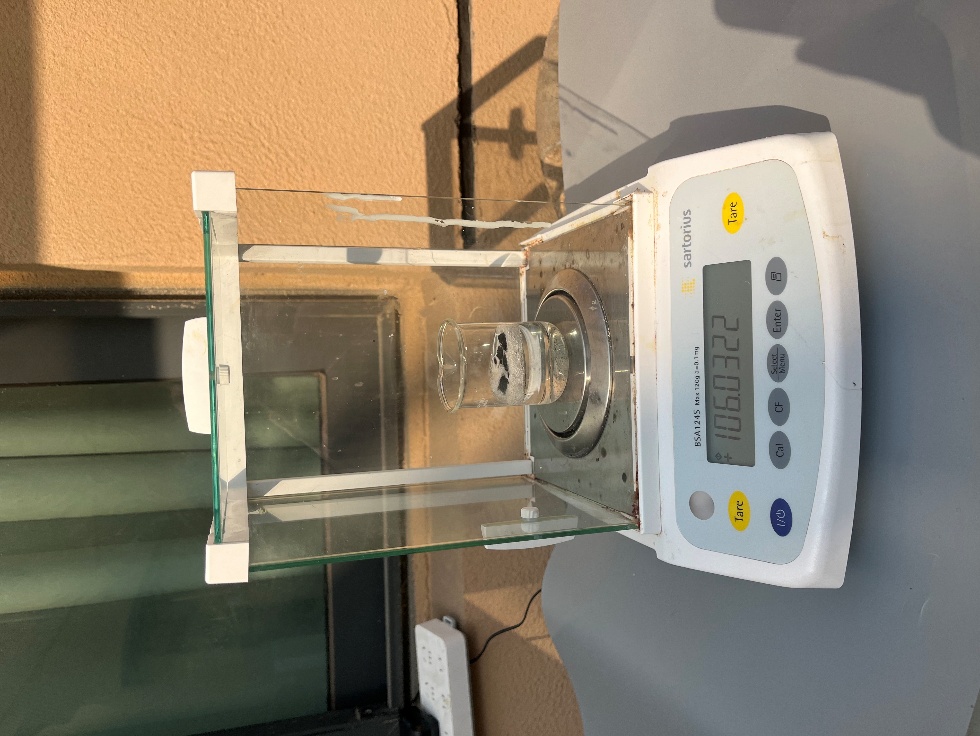


**Figure S23** Semi-sealed mass-loss measurement setup used for all quantitative evaporation experiments. All standardized indoor 1-sun tests and controlled outdoor measurements were performed using this semi-sealed configuration. In this setup, the mass loss of the water reservoir is recorded directly by the analytical balance, ensuring that the calculated evaporation rate is unaffected by condensation behavior or ambient airflow.

**
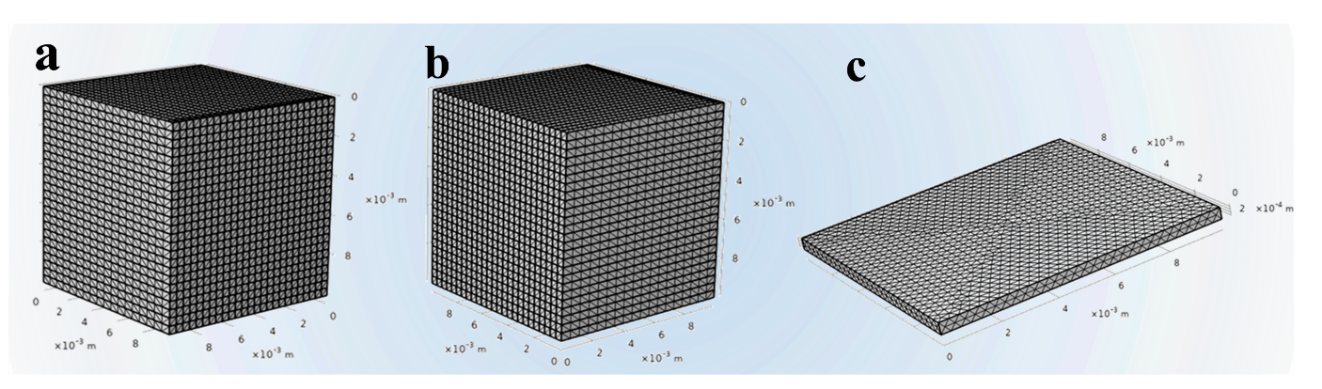
**

**Figure S24** a-b) Mesh generation for a-BTCG in-plane and BN through-plane.

The axial T_C_ (BN in-plane) was set at 1.31 W·m^−1^ K^−1^ and the transverse T_C_ (BN through-plane) was set at 0.08 W·m^−1^ K^−1^ on the basis of our experimental results. Both structures have the same boundary convection and radiation conditions to minimize their influence on thermal conduction. The bottom heat flux is 0.7 W cm^−2^.

**
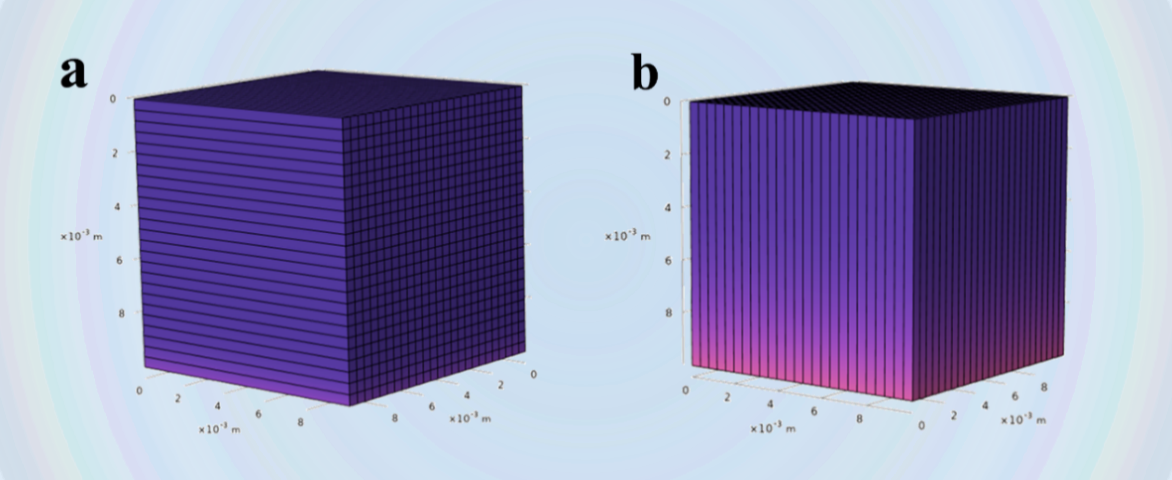
**

**Figure S25** a-b) 10 s heat transfer process for a-BTCG in-plane and BN through-plane structure.

**Table S1** Desalination performance comparison.

| **No** | **Samples** | **Characteristics** | **Stability/Energy efficiency** | **Ref.** |
| --- | --- | --- | --- | --- |
|  | **This work** | **Temperature increased from 21.3 ℃ to 36.2 ℃ within 5 min, photothermal conversion efficiency of 94.7%, the evaporation rate is 5.43 kg m^-2^ h^-1^** | **After 10 cycles (1h each), the evaporation efficiency was maintained at 90.6%** |  |
| 1 | Fe_3_O_4_ | Evaporation rate **1.3 kg m^-2^ h^-1^**, 97% strong 73light absorption over the entire wavelength range, **73%** photothermal conversion efficiency | Solar evaporation efficiency maintained at 73% for 6 h continuously | [1] |
| 2 | MoS_2_ | Evaporation rate of **1.46 kg m^-2^ h^-1^** and photothermal conversion efficiency of **82.5%** | Stabilized evaporation rate after 10 cycles of irradiation (1 hour per cycle) | [2] |
| 3 | TiO_2_@TiN | Evaporation rate **1.5252 kg m^-2^ h^-41^**, absorbs 97.42% of the sunlight, photothermal conversion efficiency **94.01%** | Stable evaporation performance over 5 cycles (15 h) | [3] |
| 4 | FeNi | Evaporation rate **1.5 kg m^-2^ h^-1^,** photothermal conversion efficiency **99.64%** | 30 cycles with stable evaporation efficiency | [4] |
| 5 | MnO_2_ | Evaporation rate **1.22 kg m^-2^ h^-1^**, photothermal conversion efficiency **81.4%** | 20 cycles with stable evaporation efficiency | [5] |
| 6 | CuFeSe_2_ | From 20°C to 51.5°C in 400 s irradiation time, photothermal conversion efficiency **86.2%** | High stable evaporation efficiency of 25 h can be maintained | [6] |
| 7 | Ti_3_C_2_ | Evaporation rate of **1.465 kg m^-2^ h^-1^** and s photothermal conversion efficiency of **96%** | Can be maintained for more than 4 cycles of more than 1h each. | [7] |
| 8 | VO_2_ | Evaporation rate **1.57 kg m^-2^ h^-1^**, photothermal conversion efficiency **93.45%** | Stable evaporation performance over 20 cycles | [8] |
| 9 | Black TiO_2_ | Evaporation rate of **2.04 kg m^-2^ h^-1^** and photothermal conversion efficiency of **90.06%** | Stable evaporation performance over 10 cycles | [9] |
| 10 | Ag_3_PO_4_ | photothermal conversion efficiency of **88.0%** and a water generation rate of **1.59 kg m^−2^ h^−1^** | Stable evaporation performance for 5 days (12 hours per day) | [10] |
| 11 | Fe-Ti_3_O_5_ | Temperature increased from 22.8 ℃ to 41.1 ℃ within 5 min, photothermal conversion efficiency of **76%,** the evaporation rate is **1.455 kg m^-2^ h^-1^** | After 3 cycles (1h each), the evaporation efficiency was maintained at 82.8% | [11] |
| 12 | PPy-carbon cloth | The evaporation rate is **2.16 kg m^2^ h^-1^** at 1 solar intensity | The enthalpy of evaporation is **1510** J g^-1^ | [12] |
| 13 | Fe_2_O_3_/N,O-doped carbon foam | The evaporation rate is **2.05 kg m^2^ h^-1^** at 1 solar intensity | The enthalpy of evaporation is **1495** J g^-1^ | [13] |
| 14 | AcC/Fe_3_O_4_@PVA/PSS hydrogel | The evaporation rate is **3.43 kg m^2^ h^-1^** at 1 solar intensity, photothermal conversion efficiency of **93.4%**. | The enthalpy of evaporation is **1031** J g^-1^ | [14] |
| 15 | C/Ag@CS/lignin hybrid aerogel | The evaporation rate is **3.569 kg m^2^ h^-1^** at 1 solar intensity, photothermal conversion efficiency of **92.05%**. | The enthalpy of evaporation is **928.5** J g^-1^ | [15] |
| 16 | SPPy@double vinyl polysiloxane aerogel | The evaporation rate is **3.61 kg m^2^ h^-1^** at 1 solar intensity, photothermal conversion efficiency of >**95%**. | The enthalpy of evaporation is **1450** J g^-1^ | [16] |
| 17 | Fe_3_O_4_/PVA-based hierarchical cellulose aerogel | The evaporation rate is **3.17 kg m^2^ h^-1^** at 1 solar intensity | The enthalpy of evaporation is **949** J g^-1^ | [17] |
| 18 | C@CuO membrane | The evaporation rate is **1.88 kg m^2^ h^-1^** at 1 solar intensity | The enthalpy of evaporation is **1502** J g^-1^ | [18] |
| 19 | Au@Ag-Pd/PS | The evaporation rate is **3.04 kg m^2^ h^-1^** at 1 solar intensity, photothermal conversion efficiency of **99.1%**. | The enthalpy of evaporation is **1340** J g^-1^ | [19] |
| 20 | MOF derived MnO/C membrane | The evaporation rate is **2.38 kg m^2^ h^-1^** at 1 solar intensity | The enthalpy of evaporation is **1680** J g^-1^ | [20] |
| 21 | NKU-123 | The evaporation rate of **1.442 and 1.299 kg m^−2^ h^−1^** under 1-sun irradiation with a water evaporation efficiency of 97.8 and 87.9 % for pure water and seawater, respectively. | | [21] |
| 22 | PVA/MNC hydrogel | The evaporation rate is **2.53 kg m^2^ h^-1^** at 1 solar intensity | The enthalpy of evaporation is **1396 J g^-1^** | [22] |
| 23 | PDA-MXene@HAP/PVA/PAM biomimetic aerogel | The evaporation rate is **2.62 kg m^2^ h^-1^** at 1 solar intensity, photothermal conversion efficiency of **93.6%**. | The enthalpy of evaporation is **1425.61** J g^-1^ | [23] |
| 24 | TiO_2_@TiN nanowires on carbonized wood | The evaporation rate is **1.5252 kg m^2^ h^-1^** at 1 solar intensity, photothermal conversion efficiency of **94.01%**. | The enthalpy of evaporation is **2151.8 J g^-1^** | [24] |
| 25 | Ti_2_O_3_@PVA sponge-like hydrogel | The evaporation rate is **3.6 kg m^2^ h^-1^** at 1 solar intensity, photothermal conversion efficiency of ~**90%**. | The enthalpy of evaporation is **912 J g^-1^** | [25] |
| 26 | MXene/rGO hybrid hydrogel | The evaporation rate is **3.62 kg m^2^ h^-1^** at 1 solar intensity, photothermal conversion efficiency of **91%**. | The enthalpy of evaporation is **905** J g^-1^ | [26] |
| 27 | PVA hydrogel | The evaporation rate is **2.6 kg m^2^ h^-1^** at 1 solar intensity, photothermal conversion efficiency of **∼91%** | The enthalpy of evaporation is **1250** J g^-1^ | [27] |
| 28 | PVA-PPy gel | The evaporation rate is **3.2 kg m^2^ h^-1^** at 1 solar intensity | The enthalpy of evaporation is **1000** J g^-1^ | [28] |
| 29 | CNTs/PVA hybrid gel | The evaporation rate is **2.06 kg m^2^ h^-1^** at 1 solar intensity, photothermal conversion efficiency of **90.05%** | The enthalpy of evaporation is **1613.9** J g^-1^ | [29] |
| 30 | Carbon dots@Balsa wood | The evaporation rate is **2.27 kg m^2^ h^-1^** at 1 solar intensity, photothermal conversion efficiency of **92.5%** | -- | [30] |
| 31 | Multilayer PPy membrane | The evaporation rate is **1.38 kg m^2^ h^-1^** at 1 solar intensity, photothermal conversion efficiency of **95.33%** | The enthalpy of evaporation is **2403** J g^-1^ | [31] |
| 32 | Ag-PSS decorated agarose gel | The evaporation rate is **2.1 kg m^2^ h^-1^** at 1 solar intensity, photothermal conversion efficiency of **92.8%** | The enthalpy of evaporation is **1591** J g^-1^ | [32] |
| 33 | Aluminum sheets | The evaporation rate is **1.5 kg m^2^ h^-1^** at 1 solar intensity | The enthalpy of evaporation is **1220** J g^-1^ | [33] |
| 34 | MoS_2_/PEI@MCE membrane | The evaporation rate is **~1 kg m^2^ h^-1^** at 1 solar intensity | The enthalpy of evaporation is **1462** J g^-1^ | [34] |
| 35 | H_x_MoO_3_/PNIPAM hydrogel | The evaporation rate is **1.65 kg m^2^ h^-1^** at 1 solar intensity, photothermal conversion efficiency of **85.87%** | The enthalpy of evaporation is **1873** J g^-1^ | [35] |
| 36 | Pani-based hierarchically porous polyion complex hydrogel | The evaporation rate is **2.79 kg m^2^ h^-1^** at 1 solar intensity | The enthalpy of evaporation is **1244** J g^-1^ | [36] |
| 37 | Poly (ionic liquid) crosslinked GO/CNT membrane | The evaporation rate is **1.87 kg m^2^ h^-1^** at 1 solar intensity | The enthalpy of evaporation is **1990** J g^-1^ | [37] |
| 38 | Vertically aligned rGO foam | The evaporation rate is **3.39 kg m^2^ h^-1^** at 1 solar intensity | The enthalpy of evaporation is **1987** J g^-1^ | [38] |
| 39 | CNTs@PVA/sodium lignosulfonate hydrogel | The evaporation rate is **2.09 kg m^2^ h^-1^** at 1 solar intensity | The enthalpy of evaporation is **1525** J g^-1^ | [39] |
| 40 | Ti_2_O_3_/PVA hydrogel | The evaporation rate is **4.0 kg m^2^ h^-1^** at 1 solar intensity, photothermal conversion efficiency of **93%** | The enthalpy of evaporation is **850 J g^-1^** | [40] |
| 41 | Vertically aligned GO/Ti_3_C_2_Tx hybrid hydrogel | The evaporation rate is **2.09 kg m^2^ h^-1^** at 1 solar intensity, photothermal conversion efficiency of **93.5%** | The enthalpy of evaporation is **1791** J g^-1^ | [41] |
| 42 | PAN@CoMn-LDH membrane | The evaporation rate is **3.12 kg m^2^ h^-1^** at 1 solar intensity | The enthalpy of evaporation is **1309** J g^-1^ | [42] |
| 43 | iCOF/cellulose membrane | The evaporation rate is **3.55 kg m^2^ h^-1^** at 1 solar intensity, photothermal conversion efficiency of **95.8%**. | The enthalpy of evaporation is **1425** J g^-1^ | [43] |
| 44 | GO-PEI membrane | The evaporation rate is **2.48 kg m^2^ h^-1^** at 1 solar intensity | The enthalpy of evaporation is **1390** J g^-1^ | [44] |
| 45 | CNT/CBs/PVA/PEI polymeric nanonetworks | The evaporation rate is **3.55 kg m^2^ h^-1^** at 1 solar intensity | The enthalpy of evaporation is **1503** J g^-1^ | [45] |
| 46 | PVA/GA/PDA-PAM hydrogel | The evaporation rate is **4.85 kg m^2^ h^-1^** at 1 solar intensity | The enthalpy of evaporation is **1135** J g^-1^ | [46] |
| 47 | PPy@alkalized loofah evaporator | The evaporation rate is **2.05 kg m^2^ h^-1^** at 1 solar intensity | The enthalpy of evaporation is **1826** J g^-1^ | [47] |
| 48 | LC@LCG  Lignocellulose-Based Hydrogel | The evaporation rate is **1.84 kg m^2^ h^-1^** at 1 solar intensity, photothermal conversion efficiency of **86.5%** | The enthalpy of evaporation is **1667** J g^-1^ | [48] |
| 49 | MOF-derived N/Co-doped carbon | The evaporation rate is **2.20 kg m^2^ h^-1^** at 1 solar intensity | The enthalpy of evaporation is **1680** J g^-1^ | [49] |
| 50 | NiV_15_@wood | The evaporation rate is **2.23 kg m^2^ h^-1^** at 1 solar intensity | The enthalpy of evaporation is **1446** J g^-1^ | [50] |
| 51 | Co-MOF/CNT membrane | The evaporation rate is **2.25 kg m^2^ h^-1^** at 1 solar intensity | The enthalpy of evaporation is **1637** J g^-1^ | [51] |
| 52 | PVA/Prussian blue hydrogel | The evaporation rate is **3.44 kg m^2^ h^-1^** at 1 solar intensity | The enthalpy of evaporation is **1126.9** J g^-1^ | [52] |
| 53 | Picosecond laser treated aluminium | The evaporation rate is **1.24 kg m^2^ h^-1^** at 1 solar intensity | The enthalpy of evaporation is **541.25** J g^-1^ | [53] |
| 54 | PVA/PEDOT:PSS hydrogel | The evaporation rate is **2.5 kg m^2^ h^-1^** at 1 solar intensity | The enthalpy of evaporation is **1624.14** J g^-1^ | [54] |
| 55 | Porous carbon-coated wood (PCW) | The evaporation rate is **2.38 kg m^2^ h^-1^** at 1 solar intensity | The enthalpy of evaporation is **1427** J g^-1^ | [55] |
| 56 | Hierarchical PAN@CuS fabric | The evaporation rate is **2.27 kg m^2^ h^-1^** at 1 solar intensity | The enthalpy of evaporation is **1956.32** J g^-1^ | [56] |
| 57 | Carbonized bamboos | The evaporation rate is **3.13 kg m^2^ h^-1^** at 1 solar intensity | The enthalpy of evaporation is **1519** J g^-1^ | [57] |
| 58 | In-air calcinated melamine sponges | The evaporation rate is **1.52 kg m^2^ h^-1^** at 1 solar intensity | The enthalpy of evaporation is **1846** J g^-1^ | [58] |
| 59 | rGO-Ag/SA@PU sponge | The evaporation rate is **2.02 kg m^2^ h^-1^** at 1 solar intensity | The enthalpy of evaporation is **1614** J g^-1^ | [59] |
| 60 | GO/CNTs-embedded origami structure | The evaporation rate is **1.59 kg m^2^ h^-1^** at 1 solar intensity | -- | [60] |
| 61 | Nitric acid-treated PVA/GO@black sponge | The evaporation rate is **2.72 kg m^2^ h^-1^** at 1 solar intensity | The enthalpy of evaporation is **1082** J g^-1^ | [61] |
| 62 | PVA/PSS hydrogel | The evaporation rate is **3.9 kg m^2^ h^-1^** at 1 solar intensity, photothermal conversion efficiency of **92%**. | The enthalpy of evaporation is **860** J g^-1^ | [62] |
| 63 | Carbon black/PVA with embossment structure | The evaporation rate is **2.15 kg m^2^ h^-1^** at 1 solar intensity | -- | [63] |
| 64 | p-toluenesulfonic acid treated wood | The evaporation rate is **2.2 kg m^2^ h^-1^** at 1 solar intensity | The enthalpy of evaporation is **1860** J g^-1^ | [64] |
| 65 | Carbon black/regenerated Cellulose | The evaporation rate is **3.01 kg m^2^ h^-1^** at 1 solar intensity | The enthalpy of evaporation is **1850** J g^-1^ | [65] |
| 66 | Porous carbon/pulp fiber (PCP-x) membranes | The evaporation rate is **1.8 kg m^2^ h^-1^** at 1 solar intensity | The enthalpy of evaporation is **1985** J g^-1^ | [66] |
| 67 | Ta_2_O_5_/C nanohollow spheres | The evaporation rate is **4.02 kg m^2^ h^-1^** at 1 solar intensity | The enthalpy of evaporation is **1491** J g^-1^ | [67] |
| 68 | MOF/CNTs membrane | The evaporation rate is **2.5 kg m^2^ h^-1^** at 1 solar intensity | The enthalpy of evaporation is **1310** J g^-1^ | [68] |
| 69 | PVA/rGO hydrogel | The evaporation rate is **2.5 kg m^2^ h^-1^** at 1 solar intensity | The enthalpy of evaporation is **1300** J g^-1^ | [69] |
| 70 | Fluorinated PPy/PVA Janus aeroge | The evaporation rate is **1.68 kg m^2^ h^-1^** at 1 solar intensity | The enthalpy of evaporation is **1710** J g^-1^ | [70] |
| 71 | MOF(SUC-700)@Wood | The evaporation rate is **2.07 kg m^2^ h^-1^** at 1 solar intensity | The enthalpy of evaporation is **1810** J g^-1^ | [71] |
| 72 | CNTs/bacterial cellulose-based hierarchical solar  composite | The evaporation rate is **2.9 kg m^2^ h^-1^** at 1 solar intensity photothermal conversion efficiency of **88%.** | The enthalpy of evaporation is **997** J g^-1^ | [72] |
| 73 | PPy/melamine foam evaporator | The evaporation rate is **2 kg m^2^ h^-1^** at 1 solar intensity | The enthalpy of evaporation is **1710** J g^-1^ | [73] |
| 74 | PDA/MXene@delignified wood | The evaporation rate is **2.08 kg m^2^ h^-1^** at 1 solar intensity | The enthalpy of evaporation is **1915** J g^-1^ | [74] |
| 75 | N,O dual-doped carbon foam (NCF) | The evaporation rate is **2.37 kg m^2^ h^-1^** at 1 solar intensity | The enthalpy of evaporation is **1524** J g^-1^ | [75] |
| 76 | PPy/PVA/CS hydrogel | The evaporation rate is **3.2 kg m^2^ h^-1^** at 1 solar intensity, photothermal conversion efficiency of **94%**. | The enthalpy of evaporation is **881** J g^-1^ | [76] |
| 77 | MXene/LSC@PVA/Chitosan hydrogel | The evaporation rate is **2.73 kg m^2^ h^-1^** at 1 solar intensity | The enthalpy of evaporation is **1175** J g^-1^ | [77] |

The efficiency calculation of photothermal conversion involves the following calculation formula:

$\Delta m=\frac{dm}{Adt}$ (1)

Where ∆*m* is the evaporation rate, and m is the mass change; *A* is the surface area of the photothermal material; *t* is time.

$\eta_{vapor}=\frac{\Delta m\cdot h}{Q\cdot A}$ (2)

Where *η* is the vapor conversion efficiency, *h* is the latent heat required for water evaporation at standard atmospheric pressure (2.257 kJ/kg), *Q* is the intensity of incident light.

$\eta=\eta_{water}+\eta_{vapor}=\frac{Cm\Delta T}{Q\cdot A}+\frac{\Delta m\cdot h}{Q\cdot A}$ (3)

The photothermal conversion efficiency (*η*) includes both *η*_vapor_ and *η*_water_. Where *C* is the specific heat capacity of water, and ∆*T* is the increased temperature.

**Table S2** Thermal-responsive performance comparison.

| **Types** | **Working mechanism** | **Response temperature** | **Fire trigger time** | **Recyclability** | |  | **Ref.** |
| --- | --- | --- | --- | --- | --- | --- | --- |
| **This work** | **Semiconductor properties** | **170 ºC** | **0.42 s** | | **Yes** |  |  |
| PGO@HN/GF paper | Carbon-based nanomaterials | 126.9 °C | 2 s | | No |  | [78] |
| BP‑MoS_2_/GO film | Carbon-based nanomaterials | 500 °C | 1 s | | No |  | [79] |
| GO/LAA/silane | Carbon-based nanomaterials | 100-300 °C | 0.93 s | | No |  | [80] |
| VGO-CB bilayer film | Carbon-based nanomaterials | - | 0.2 s | | No |  | [81] |
| MMT/chitosan/A‑CNT aerogel | Carbon‑based nanomaterials | - | 0.25 s | | Yes |  | [82] |
| TA-GO/HHACP | Carbon‑based nanomaterials | 200 ℃ | 0.6 s | | NO |  | [83] |
| GO/PA/PVA | Carbon‑based nanomaterials | 150 ℃ | 2 s | | NO |  | [84] |
| MXene@V/SA | Semiconductor properties | 500 ℃ | 3.6 s | | Yes |  | [85] |
| AgNW@Fe_3_O_4_ | Semiconductor properties | 100 ℃ | 2 s | | Yes |  | [86] |
| CB@KF‑PVA‑CNT | Semiconductor properties | 350 ℃ | 4 s | | Yes |  | [87] |
| ANF/PEG/Fe_3_O_4_ NW/PANI | Semiconductor properties | 100 ℃ | 1.3 s | | Yes |  | [88] |
| PA/C-MXene-coatings | Semiconductor properties | - | 2.1 s | | Yes |  | [89] |
| PEI/APP/Ti_3_O_5_-coatings | Semiconductor properties | 190℃ | 3.78 s | | Yes |  | [90] |
| ZnS | Color change | 260 ℃ | 0.9 s | | Yes |  | [91] |
| PMS | Color change | 275 °C | 20 s | | No |  | [92] |
| PCL/Ag layer | Shape memory | 45 ºC | 5 s | | No |  | [93] |
| N-type Ti_3_C_2_T_x_ MXene and P-type MXene/SWCNT-COOH | Thermoelectric effect | 100 ℃ | 1.43 s | | Yes |  | [94] |
| Ag_2_Se/AgNW/PVB | Thermoelectric effect | 100-300 ℃ | 2 s | | Yes |  | [95] |
| CA/Fe_3_O_4_ NPs, and AgNWs | Thermoelectric/Triboelectric | 100-400 ℃ | 2 s | | Yes |  | [96] |
| TEG/CCS | Thermoelectric effect | 50-350 ℃ | 1.5 s | | Yes |  | [97] |

**Table S3** Simulation parameters and boundary conditions.

| Size of single  filament | Length (mm) | Width (mm) | | Height (mm) |
| --- | --- | --- | --- | --- |
|  | 10 | 0.4 | | 0.4 |
| Thermal  conductivity (W m^-1^ K^-1^) | 2.7371 | | 0.4714 | |
| Convection  coefficient (W m^-2^ K^-1^) | 3 | | | |
| Bottom heat  power density (W cm^-2^) | 0.7 | | | |
| Specific heat  capacity（J kg^-1^ ℃^-1^） | 2900 | | | |
| Density（kg m^-3^） | 1082.4 | | | |
| Initial  temperature | 293 K | | | |

The simulation models were built by imitating the real 3D printed structures. The average temperature is used to evaluate the comprehensive thermal conduction properties of these two models.

**Table S4.** Comparison of temperature rise rate in representative solar-driven interfacial evaporators

| **Material System** | **ΔT (°C) within 5–20 min** | **Time to steady state** | **Ref.** |
| --- | --- | --- | --- |
| BTCG | +13.1°C (5 min) | 5 min | This work |
| Wood–TiO₂/TiN evaporator | ~12°C (15 min) | 12–15 min | [98] |
| MXene hydrogel (Ti₃C₂Tx) | ~10°C (15–20 min) | 8 min | [99] |
| 3D-printed cellulose aerogel | ~14°C (20 min) | 20–25 min | [100] |
| rGO Foam | 9–10°C | 15 min | [101] |

**References:**

[1] Song L.; Zhang X.; Wang Z.; Zheng T.; Yao J. Fe_3_O_4_/Polyvinyl Alcohol Decorated Delignified

Wood Evaporator for Continuous Solar Steam Generation. *Desalination.* **2021,** 507, 115024.

[2] He X.; Zhang L.; Hu X.; Zhou Q. Formation of S defects in MoS_2_-Coated Wood for High-Efficiency Seawater Desalination. *Environmental Science: Nano.* **2021,** 8(7), 2069-2080.

[3] Ren P.; Li J.; Zhang X.; Yang X. Highly Efficient Solarss Water Evaporation of TiO_2_@TiN Hyperbranched Nanowires-Carbonized Wood Hierarchical Photothermal Conversion Material. *Materials Today Energy****.* 2020,** 18, 100546.

[4] Mehrkhah R.; Goharshadi E. K.; Mohammadi M. Highly Efficient Solar Desalination and Wastewater Treatment by Economical Wood-Based Double-Layer Photoabsorbers. *Journal of Industrial and Engineering Chemistry.* **2021,** 101, 334-347.

[5] Li D.; Han D.; Guo C.; Huang C. Facile Preparation of MnO_2_-Deposited Wood for High-Efficiency Solar Steam Generation. *ACS Applied Energy Materials.* **2021,** 4(2), 1752-1762.

[6] Liu H.; Chen C.; Wen H.; Guo R.; Williams N. A.; Wang B.; Chen F.; Hu L. Narrow Bandgap Semiconductor Decorated Wood Membrane for High-Efficiency Solar-Assisted Water Purification. *Journal of Materials Chemistry A.* **2018,** 6(39), 18839-18846.

[7] Ma N.; Fu Q.; Hong Y.; Hao X.; Wang X.; Ju J.; Sun J. Processing Natural Wood into an Efficient and Durable Solar Steam Generation Device. *ACS Appl. Mater. Interfaces.* **2020,** 12(15), 18165-18173.

[8] Aziznezhad M.; Goharshadi E. K.; Mehrkhah R.; Ghafurian M. M. Alkaline Earth Metals Doped VO_2_ Nanoparticles for Enhanced Interfacial Solar Steam Generation. *Materials Research Bulletin.* **2022,** 149, 111705.

[9] Xiao B.; Yu F.; Xia Y.; Wang J.; Xiong X.; Wang X. Wood-Based, Bifunctional, Mulberry-Like Nanostructured Black Titania Evaporator for Solar-Driven Clean Water Generation. *Energy Technology.* **2022,** 10(3), 2100679.

[10] Xi Y.; Du C.; Li P.; Zhou X.; Zhou C.; Yang S. Combination of Photothermal Conversion and Photocatalysis toward Water Purification. *Industrial & Engineering Chemistry Research.* **2022,** 61(13), 4579-4587.

[11] Sun S.; Liu C.; Zhang S.; Wu Q.; Tian D.; Mei C.; Pan M. Rich Oxygen Vacancies Mediated Metal-Insulator Transition Materials toward Ultrasensitive Sensing and Energy Conversion. *Nano Energy*. **2024,** 126, 109606.

[12] Yu Z.; Gu R.; Tian Y.; Xie P.; Jin B.; Cheng S. Enhanced Interfacial Solar Evaporation through Formation of Micro-Meniscuses and Microdroplets to Reduce Evaporation Enthalpy. *Adv. Funct. Mater.* **2022**, 32(17), 2108586.

[13] Bai H.; He P.; Hao L.; Liu N.; Fan Z.; Chen B.; Niu R.; Gong J. Engineering Self-Floating Fe_2_O_3_/N, O-Doped Carbon Foam as a Bifunctional Interfacial Solar Evaporator for Synergetic Freshwater Production and Advanced Oxidation Process. *J. Environ. Chem. Eng.* 2022, 10(5), 108338.

[14] Wang B.; Yang K.; Cai B.; Zhang J.; Wei C.; Zhou A. A Magnetic Nanostructure PAC@Fe_3_O_4_ Driven Design toward Janus Hydrogel Achieves Highly Efficient Solar Water Evaporation. *J. Chem. Eng.* **2023**, 465, 142944.

[15] Chen S.; Yoo C.; Yang D.; Qiu X.; Zheng D. Multifunctional Lignin-Mediated Biomass Hybrid Aerogel with Plasmon-Enhanced Solar-Driven Desalination and Sewage Purification. *Desalination* **2023**, 556, 116572.

[16] Zhao X.; Wang T.; Jiang Y.; Lu Q.; Pan J. Robust and Versatile Polypyrrole Supramolecular Network Packed Photothermal Aerogel for Solar-Powered Desalination. *Desalination* **2023**, 561, 116674.

[17] Sun J.; Teng R.; Tan J.; Xu M.; Ma C.; Li W.; Liu S. An Integrated Cellulose Aerogel Evaporator with Improved Thermal Management and Reduced Enthalpy of Evaporation Using a Hierarchical Coordinated Control Strategy. *J. Mater. Chem. A* **2023**, 11(12), 6248.

[18] Hou L.; Wang N.; Yu L.-J.; Liu J.; Zhang S.; Cui Z.; Li S.; Li H.; Liu X.; Jiang L.; Zhao Y. High-Performance Janus Solar Evaporator for Water Purification with Broad Spectrum Absorption and Ultralow Heat Loss. *ACS Energy Lett.* **2022**, 8, 553-564.

[19] Chen Z.; Wang J.; Zhou H.; Xie Z.; Shao L.; Chen A.; Wang S. B.; Jiang N. Janus Nano-Micro Structure-Enabled Coupling of Photothermal Conversion, Heat Localization and Water Supply for High-Efficiency Solar-Driven Interfacial Evaporation. *Adv. Funct. Mater.* **2023**, 33(41), 2303656.

[20] Fan Z.; Ren J.; Bai H.; He P.; Hao L.; Liu N.; Chen B.; Niu R.; Gong J. Shape-Controlled Fabrication of MnO/C Hybrid Nanoparticle from Waste Polyester for Solar Evaporation and Thermoelectricity Generation. *J. Chem. Eng.* **2023**, 451, 138534.

[21] Lan W.; Gou X.; Wu Y.; Niu N.; Lu L.; Cheng P.; Shi W. The Influence of Light-Generated Radicals for Highly Efficient Solar-Thermal Conversion in an Ultra-Stable 2D Metal-Organic Assembly. *Angewandte International Edit. Chemie,* **2024,** 63(20), e202401766.

[22] Zou H.; Meng X.; Zhao X.; Qiu J. Hofmeister Effect-Enhanced Hydration Chemistry of Hydrogel for High-Efficiency Solar-Driven Interfacial Desalination. *Adv. Mater.* **2023**, 35(5), 2207262.

[23] Wang Z.-Y.; Zhu Y.-J.; Chen Y.-Q.; Yu H.-P.; Xiong Z.-C. Bioinspired Aerogel with Vertically Ordered Channels and Low Water Evaporation Enthalpy for High-Efficiency Salt-Rejecting Solar Seawater Desalination and Wastewater Purification. *Small* **2023**, 19(19), 2206917.

[24] Ren P.; Li J.; Zhang X.; Yang X.; Highly Efficient Solar Water Evaporation of TiO_2_@Tin Hyperbranched Nanowires-Carbonized Wood Hierarchical Photothermal Conversion Material. *Mater. Today Energy* **2020,** 18, 100546.

[25] Guo Y.; Zhou X.; Zhao F.; Bae J.; Rosenberger B.; Yu G. Synergistic Energy Nanoconfinement and Water Activation in Hydrogels for Efficient Solar Water Desalination. *ACS Nano* **2019,** 13(7), 7913-7919.

[26] Lu Y.; Fan D.; Wang Y.; Xu H.; Lu C.; Yang X. Surface Patterning of Two-Dimensional Nanostructure-Embedded Photothermal Hydrogels for High-Yield Solar Steam Generation. *ACS Nano* **2021,** 15, 10366-10376.

[27] Guo Y.; Zhao F.; Zhou X.; Chen Z.; Yu G. Tailoring Nanoscale Surface Topography of Hydrogel for Efficient Solar Vapor Generation. *Nano Lett.* **2019**, 19(4), 2530-2536.

[28] Zhou X.; Zhao F.; Guo Y.; Rosenberger B.; Yu G. Architecting Highly Hydratable Polymer Networks to Tune the Water State for Solar Water Purification. *Sci. Adv.* **2019,** 5(6), eaaw5484.

[29] Hu G.; Cao Y.; Huang M.; Wu Q.; Zhang K.; Lai X.; Tu J.; Tian C.; Liu J.; Huang W.; Ding L. Salt-Resistant Carbon Nanotubes/Polyvinyl Alcohol Hybrid Gels with Tunable Water Transport for High-Efficiency and Long-Term Solar Steam Generation. *Energy Technol.* **2020,** 8(1), 1900721.

[30] Hou Q.; Xue C.; Li N.; Wang H.; Chang Q.; Liu H.; Yang J.; Hu S. Self-Assembly Carbon Dots for Powerful Solar Water Evaporation. *Carbon* **2019,** 149, 556-563.

[31] Wang X.; Liu Q.; Wu S.; Xu B.; Xu H. Multilayer Polypyrrole Nanosheets with Self-Organized Surface Structures for Flexible and Efficient Solar-Thermal Energy Conversion. *Adv. Mater.* **2019**, 31(19), 1807716.

[32] Sun Z.; Wang J.; Wu Q.; Wang Z.; Wang Z.; Sun J.; Liu C.-J. Plasmon Based Double-Layer Hydrogel Device for a Highly Efficient Solar Vapor Generation. *Adv. Funct. Mater.* **2019**, 29(29), 1901312.

[33] Singh S. C.; ElKabbash M.; Li Z.; Li X.; Regmi B.; Madsen M.; Jalil S. A.; Zhan Z.; Zhang J.; Guo C. Solar-Trackable Super-Wicking Black Metal Panel for Photothermal Water Sanitation. *Nat. Sustain.* **2020**, 3, 938-946.

[34] Li Y.; Zhao M.; Xu Y.; Chen L.; Jiang T.; Jiang W.; Yang S.; Wang Y. Manipulating Light Trapping and Water Vaporization Enthalpy via Porous Hybrid Nanohydrogels for Enhanced Solar-Driven Interfacial Water Evaporation with Antibacterial Ability. *J. Mater. Chem. A* **2019**, 7(47), 26769.

[35] Cao S.; Jiang J.; Tian Q.; Guo C.; Wang X. Dai K.; Xu Q. Building of Multifunctional and Hierarchical HxMoO_3_/PNIPAM Hydrogel for High-Efficiency Solar Vapor Generation. *Green Energy Environ.* **2022**, 7(5), 1006-1013.

[36] Zhu F.; Wang L.; Demir B.; An M.; Wu Z.-L.; Yin J.; Xiao R.; Zheng Q.; Qian J. Accelerating Solar Desalination in Brine Through Ion Activated Hierarchically Porous Polyion Complex Hydrogels. *Mater. Horiz.* **2020,** 7(12), 3187.

[37] Han J.; Dong Z.; Hao L.; Gong J.; Zhao Q. Poly (Ionic Liquid)-Crosslinked Graphene Oxide/Carbon Nanotube Membranes as Efficient Solar Steam Generators. *Green Energy Environ.* **2021**, 8, 151.

[38] Li W.; Tian X.; Li X.; Han S.; Li C.; Zhai X.-Z.; Kang Y.; Yu Z.-Z. Ultrahigh Solar Steam Generation Rate of a Vertically Aligned Reduced Graphene Oxide Foam Realized by Dynamic Compression. *J. Mater. Chem. A* **2021**, 9(26), 14859.

[39] Hao L.; Liu N.; Bai H.; He P.; Niu R.; Gong J. High-Performance Solar-Driven Interfacial Evaporation through Molecular Design of Antibacterial, Biomass-Derived Hydrogels. *J. Colloid Interface Sci.* **2022**, 608, 840-852.

[40] Guo Y.; Zhao X.; Zhao F.; Jiao Z.; Zhou X.; Yu G. Tailoring Surface Wetting States for Ultrafast Solar-Driven Water Evaporation. *Energy Environ. Sci.* **2020**, 13(7), 2087.

[41] Li W.; Li X.; Chang W.; Wu J.; Liu P.; Wang J.; Yao X.; Yu Z.-Z. Vertically Aligned Reduced Graphene Oxide/Ti_3_C_2_T_x_ Mxene Hybrid Hydrogel for Highly Efficient Solar Steam Generation. *Nano Res.* **2020**, 13, 3048-3056.

[42] Ren P.; Li J.; Zhang X.; Yang X. Highly Efficient Solar Water Evaporation of TiO_2_@TiN Hyperbranched Nanowires-Carbonized Wood Hierarchical Photothermal Conversion Material. *Mater. Today Energy* **2020**, 18, 100546.

[43] Li G.; Yue Q.; Fu P.; Wang K.; Zhou Y.; Wang J. Ionic Dye Based Covalent Organic Frameworks for Photothermal Water Evaporation. *Adv. Funct. Mater.* **2023**, 33(34), 2213810.

[44] Su Y.; Liu L.; Gao X.; Yu W.; Hong Y.; Liu C. A High-Efficient and Salt-Rejecting 2D Film for Photothermal Evaporation. *iScience* **2023**, 26(8), 107347.

[45] Zhao L.; Yang Z.; Wang J.; Zhou Y.; Cao P.; Zhang J.; Yuan P.; Zhang Y.; Li Q. Boosting Solar-Powered Interfacial Water Evaporation by Architecting 3D Interconnected Polymetric Network in CNT Cellular Structure. *J. Chem. Eng.* **2023**, 451, 138676.

[46] Zhou J.; Sun Z.; Mu X.; Zhang J.; Wang P.; Chen Y.; Wang X.; Gao J.; Miao L.; Sun L. Highly Efficient and Long-Term Stable Solar-Driven Water Purification through a Rechargeable Hydrogel Evaporator. *Desalination* **2022**, 537, 115872.

[47] Wang J.; Chen Z.; Feng L.; Yu F.; Ran C.; Xu N.; Jia Z.; Li C.; Zheng Y.; Shi W.; Li M. Plants Transpiration-Inspired Antibacterial Evaporator with Multiscale Structure and Low Vaporization Enthalpy for Solar Steam Gneration. *Nano Energy* **2023**, 108631.

[48] Lin X.; Wang P.; Hong R.; Zhu X.; Liu Y.; Pan X.; Qiu X.; Qin Y. Fully Lignocellulosic Biomass-Based Double-Layered Porous Hydrogel for Efficient Solar Steam Generation. *Adv. Funct. Mater.* **2022**, 32(51), 2209262.

[49] He P.; Bai H.; Fan Z.; Hao L.; Liu N.; Chen B.; Niu R.; Gong J. Controllable Synthesis of N/Co-Doped Carbon from Metal-Organic Frameworks for Integrated Solar Vapor Generation and Advanced Oxidation Processes. *J. Mater. Chem. A* **2022,** 10(25), 13378.

[50] Zhang T.; Yan W.; Wang Y.; Wang J.; Liu C.; Ye F.; Liu B. An Ecofriendly and Efficient Wood-Based Polyoxovanadate Solar Evaporation Generator. *Sci. China Mater.* **2023**, 66, 3292-3299.

[51] Bai H.; He P.; Hao L.; Fan Z.; Niu R.; Tang T.; Gong J. Waste-Treating-Waste: Upcycling Discarded Polyester into Metal-Organic Framework Nanorod for Synergistic Interfacial Solar Evaporation and Sulfate-Based Advanced Oxidation Process. *J. Chem. Eng.* **2023**, 456, 140994.

[52] Wang W.; Wang Y.; Zheng J.; Yu X.; Chen W.; Li J.; Liu Y.-N. A Vasculatural Hydrogel Combined with Prussian Blue for Solar-Driven Vapor Generation. *J. Mater. Chem. A* **2022**, 10(23), 12608.

[53] Chen Z.; Lin Y.; Qian Q.; Su P.; Ding Y.; Tuan P. D.; Chen L.; Feng D. Picosecond Laser Treated Aluminium Surface for Photothermal Seawater Desalination. *Desalination* **2022**, 528, 115561.

[54] Li C.; Zhu B.; Liu Z.; Zhao J.; Meng R.; Zhang L.; Chen Z. Polyelectrolyte-Based Photothermal Hydrogel with Low Evaporation Enthalpy for Solar-Driven Salt-Tolerant Desalination. *J. Chem. Eng.* **2022**, 431, 134224.

[55] Liu N.; Hao L.; Zhang B.; Niu R.; Gong J.; Tang T. Rational Design of High-Performance Bilayer Solar Evaporator by Using Waste Polyester-Derived Porous Carbon-Coated Wood. *Energy Environ. Mater.* **2022**, 5(2), 617-626.

[56] Liu Z.; Zhou Z.; Wu N.; Zhang R.; Zhu B.; Jin H.; Zhang Y.; Zhu M.; Chen Z. Hierarchical Photothermal Fabrics with Low Evaporation Enthalpy as Heliotropic Evaporators for Efficient, Continuous, Salt-Free Desalination. *ACS Nano* **2021**, 15(8), 13007-13018.

[57] Bian Y.; Du Q.; Tang K.; Shen Y.; Hao L.; Zhou D.; Wang X.; Xu Z.; Zhang H.; Zhao L.; Zhu S.; Ye J.; Lu H.; Yang Y.; Zhang R.; Zheng Y.; Gu S. Carbonized Bamboos as Excellent 3D Solar Vapor-Generation Devices. *Adv. Mater. Technol.* **2019**, 4(4), 1800593.

[58] Gong F.; Li H.; Wang W.; Huang J.; Xia D.; Liao J.; Wu M.; Papavassiliou D. V. Scalable, Eco-Friendly and Ultrafast Solar Steam Generators Based on One-Step Melamine-Derived Carbon Sponges toward Water Purification. *Nano Energy* **2019**, 58, 322-330.

[59] Liu C.; Cai C.; Ma F.; Zhao X.; Ahmad H. Accelerated Solar Steam Generation for Efficient Ions Removal. *J. Colloid Interface Sci.* **2020**, 560, 103-110.

[60] Hong S.; Shi Y.; Li R.; Zhang C.; Jin Y.; Wang P. Nature-Inspired, 3D Origami Solar Steam Generator toward Near Full Utilization of Solar Energy. *ACS Appl. Mater. Interfaces* **2018**, 10(34), 28517-28524.

[61] Bai X.; Li Y.; Zhang F.; Xu Y.; Wang S.; Fu G. Mass Production of Superhydrophilic Sponges for Efficient and Stable Solar-Driven Highly Corrosive Water Evaporation. *Environ. Sci. Water Res. Technol.* **2019**, *5*, 2041.

[62] Zhou X.; Guo Y.; Zhao F.; Shi W.; Yu G. Topology-Controlled Hydration of Polymer Network in Hydrogels for Solar-Driven Wastewater Treatment. *Adv. Mater.* **2020**, *32*, 2007012.

[63] Deng Z.; Miao L.; Liu P.-F.; Zhou J.; Wang P.; Gu Y.; Wang X.; Cai H.; Sun L.; Tanemura S. Extremely High Water-Production Created by A Nanoink-Stained PVA Evaporator with Embossment Structure. *Nano Energy* **2019**, *55*, 368.

[64] Zhang X.; Yang L.; Dang B.; Tao J.; Li S.; Zhao S.; Li W.; Li J.; Chen Z.; Liu S. Nature-Inspired Design: *p-*toluenesulfonic Acid-Assisted Hydrothermally Engineered Wood for Solar Steam Generation. *Nano Energy* **2020**, *78*, 105322.

[65] Koh J. J.; Lim G. J.; Chakraborty S.; Zhang Y.; Liu S.; Zhang X.; Tan S. C.; Lyu Z.; Ding J.; He C. Robust, 3D-Printed Hydratable Plastics for Effective Solar Desalination. *Nano Energy* **2021**, 79, 105436.

[66] Hao L.; Liu N.; Niu R.; Gong J.; Tang T. High-Performance Salt-Resistant Solar Interfacial Evaporation by Flexible Robust Porous Carbon/Pulp Fiber Membrane. *Sci. China Mater.* **2022**, *65*, 201.

[67] Chen X.; Yang N.; Wang Y.; He H.; Wang J.; Wan J.; Jiang H.; Xu B.; Wang L.; Yu R.; Tong L.; Gu L.; Xiong Q.; Chen C.; Zhang S.; Wang D. Highly Efficient Photothermal Conversion and Water Transport During Solar Evaporation Enabled by Amorphous Hollow Multishelled Nanocomposites. *Adv. Mater.* **2022**, 34, 2107400.

[68] Dong Z.; Zhang C.; Peng H.; Gong J.; Zhao Q. Modular Design of Solar-Thermal Nanofluidics for Advanced Desalination Membranes. *J. Mater. Chem. A* **2020**, 8, 24493-24500.

[69] Zhou X.; Zhao F.; Guo Y.; Zhang Y.; Yu G. A Hydrogel-based Antifouling Solar Evaporator for Highly Efficient Water Desalination. *Energy Environ. Sci.* **2018**, *11*, 1985.

[70] Wen B.; Zhang X.; Yan Y.; Huang Y.; Lin S.; ZhuY.; Wang Z.; Zhou B.; Yang S.; Liu J. Tailoring Polypyrrole-Based Janus Aerogel for Efficient and Stable Solar Steam Generation. *Desalination* **2021**, *516*, 115228.

[71] He P.; Hao L.; Liu N.; Bai H.; Niu R.; Gong sJ. Controllable Synthesis of Sea Urchin-Like Carbon from Metal-Organic Frameworks for Advanced Solar Vapor Generators. *Chem. Eng. J.* **2021**, 423, 130268.

[72] Guan Q.-F.; Han Z.-M.; Ling Z.-C.; Yang H.-B.; Yu S.-H. Sustainable Wood-Based Hierarchical Solar Steam Generator: A Biomimetic Design with Reduced Vaporization Enthalpy of Water. *Nano Lett.* **2020**, *20*, 5699.

[73] Chen J.; Li B.; Hu G.; Aleisa R.; Lei S.; Yang F.; Liu D.; Lyu F.; Wang M; Ge X.; Qian F.; Zhang Q.; Yin Y. Integrated Evaporator for Efficient Solar-Driven Interfacial Steam Generation. *Nano Lett.* **2020**, *20*, 6051-6058.

[74] Chen Y.; Yang J.; Zhu L.; Jia X.; Wang S.; Li Y.; Song H. An Integrated Highly Hydrated Cellulose Network with A Synergistic Photothermal Effect for Efficient Solar-Driven Water Evaporation and Salt Resistance. *J. Mater. Chem. A* **2021**, *9*, 15482.

[75] Bai H.; Liu N.; Hao L.; He P.; Ma C.; Niu R.; Gong J.; Tang T. Self-Floating Efficient Solar Steam Generators Constructed Using Super-Hydrophilic N,O Dual-Doped Carbon Foams from Waste Polyester. *Energy Environ. Mater.* **2022**, *5*, 1204.

[76] Zhao F.; Zhou X.; Shi Y.; Qian X.; Alexander M.; Zhao X.; Mendez S.; Yang R.; Qu L.; Yu G. Highly Efficient Solar Vapour Generation via Hierarchically Nanostructured Gels. *Nat. Nanotechnol.* **2018**, 13, 489.

[77] Fan D.; Lu Y.; Zhang H.; Xu H.; Lu C.; Tang Y.; Yang X. Synergy of Photocatalysis and Photothermal Effect in Integrated 0D Perovskite Oxide/2D MXene Heterostructures for Simultaneous Water Purification and Solar Steam Generation. *Appl. Catal. B: Environ.* **2021**, 295, 120285.

[78] Chen F.-F.; Zhu Y.-J.; Chen F.; Dong L.-Y.; Yang R.-L.; Xiong Z.-C. Fire Alarm Wallpaper Based on Fire-Resistant Hydroxyapatite Nanowire Inorganic Paper and Graphene Oxide Thermosensitive Sensor. *ACS Nano.* **2018,** 12(4), 3159-71.

[79] Qu Z.; Xu C.; Li X.; Wu Y.; Wang K.; Zheng X.; Cui X.; Wu X.; Shi J.; Wu K. Facile Preparation of BP-MoS_2_/GO Composite Films with Excellent Flame Retardancy and Ultrasensitive Response for Smart Fire Alarm. *Chem. Eng. J.* **2021,** 426, 130717.

[80] Zhang Z.-H.; Zhang J.-W.; Cao C.-F.; Guo K.-Y.; Zhao L.; Zhang G.-D.; Gao J.-F.; Tang L.-C. Temperature-Responsive Resistance Sensitivity Controlled by L-ascorbic Acid and Silane Co-Functionalization in Flame-Retardant GO Network for Efficient Fire Early-Warning Response. *Chem. Eng. J.* **2020,** 386, 123894.

[81] Xiao D.; Zheng M.; Wu F. A Bio-Inspired Self-Assembled Asymmetrical Supramolecular Film for Highly-Sensitive Fire Warning, Solvent Response, and Smart Switching. *Chem. Eng. J.* **2023,** 459.

[82] Chen J, Xie H, Lai X, Li H, Gao J, Zeng X. An Ultrasensitive Fire-Warning Chitosan/Montmorillonite/Carbon Nanotube Composite Aerogel with High Fire-Resistance. *Chem. Eng. J.* **2020,** 399, 125729.

[83] Zhang P.; Wang Y.; Mao C.; Mao S.; Peng J.; Zhang L.; Wang Y.; Xu P.; Luo Y.; Chen A.-Z. Mechanically Flexible Graphene Oxide Network for Highly-Sensitive and Ultra-Long Fire Warning. *Chem. Eng. J.* **2024**, 494, 153163.

[84] Xi J.; Lou Y.; Meng L.; Deng C.; Chu Y.; Xu Z.; Xiao H.; Wu W. Smart Cellulose-Based Janus Fabrics with Switchable Liquid Transportation for Personal Moisture and Thermal Management. *Nano-Micro Lett*. **2025**, 17,14.

[85] Wang K.; Wang Y.; Yao A.; Hu H.; Fan W.; Lin S.; Lan J. A Natural Clay-based Janus Micro-Nanosystem with Ultra-Temperature Resistant Piezoresistive Sensing and Self-Switching Fire Warning for Smart Fire Safety. *Chem. Eng. J.* **2025**, 505, 159483.

[86] Zhang M.; Wang M.; Zhang M.; Yang C.; Li Y.; Zhang Y.; Hu J.; Wu G. Flexible and Thermally Induced Switchable Fire Alarm Fabric Based On Layer-by-Layer Self-Assembled Silver Sheet/Fe3O4 Nanowire Composite. *ACS Appl. Mater. Interfaces.* **2019,** 11(50), 47456-67.

[87] Xia L.; Lv Y.; Miao Z.; Luo L.; Luo W.; Xu Y.; Yuan C.; Zeng B.; Dai L. A Flame Retardant Fabric Nanocoating Based on Nanocarbon Black Particles@Polymer Composite and its Fire-Alarm Application. *Chem. Eng. J.* **2022,** 433, 133501.

[88] Yu Z.; Wan Y.; Qin Y.; Jiang Q.; Guan J.-P.; Cheng X.-W.; Wang X.; Ouyang S.; Qu X.; Zhu Z.; Wang J.; He H. High Fire Safety Thermal Protective Composite Aerogel with Efficient Thermal Insulation and Reversible Fire Warning Performance for Firefighting Clothing. *Chem. Eng. J.* **2023,** 477, 147187.

[89] Zhang Y.; Huang Y.; Li M.-C.; Zhang S.; Zhou W.; Mei C.; Pan M. Bioinspired, Stable Adhesive Ti_3_C_2_Tx MXene-based Coatings towards Fire Warning, Smoke Suppression and VOCs Removal Smart Wood. *Chem. Eng. J.* **2023,** 452(4), 139360.

[90] Zhang S.; Zhang Y.; Huang Y.; Lin B.; Ling S.; Mei C.; Pan M. Intelligent Coating Based on Metal-Insulator Transitional Ti_3_O_5_ towards Fire Sensing and Protection. *Chem. Eng. J.* **2022,** 450, 137910.

[91] Shi Q.; Liang J.; Wang X.; Yao K.; Tang Y.; Pan C.; Sun J.; Liu R.; Tan H.; Tang T. Chameleon Inspired High-Temperature Thermochromic Traffic Light Type Photonic Crystal Sensors toward Early Fire Detection and Visual Sensing. *Chem. Eng. J.* **2024**, 485, 149966.

[92] Fu T.; Zhao X.; Chen L.; Wu W.-S.; Zhao Q.; Wang X.-L.; Guo D.-M.; Wang Y.-Z. Bioinspired Color Changing Molecular Sensor toward Early Fire Detection Based on Transformation of Phthalonitrile to Phthalocyanine. *Adv. Funct. Mater.* **2019,** 29(8), 1806586.

[93] Jia J.; Gao N.; Li R.; Liao S.; Lyu S.; Wang Y. An “OFF-to-ON” Shape Memory Polymer Conductor for Early Fire Disaster Alarming. *Chem. Eng. J.* **2022,** 431, 133285.

[94] He H.; Qin Y.; Zhu Z.; Jiang Q.; Ouyang S.; Wan Y.; Qu X.; Xu J.; Yu Z. Temperature-Arousing Self-Powered Fire Warning E-Textile Based on *p-n* Segment Coaxial Aerogel Fibers for Active Fire Protection in Firefighting Clothing. *Nano-Micro Letters.* **2023,** 15, 226.

[95] Xie H, Lai X, Li H, Gao J, Zeng X. Skin-Inspired Thermoelectric Nanocoating for Temperature-Sensing and Fire Safety. *J Colloid Interface Sci.* **2021,** 602, 756-66.

[96] He H.; Liu J.; Wang Y.; Zhao Y.; Qin Y.; Zhu Z.; Yu Z.; Wang J. An Ultralight Self-Powered Fire Alarm e-Textile Based on Conductive Aerogel Fiber with Repeatable Temperature Monitoring Performance Used in Firefighting Clothing. *ACS Nano.* **2022,** 16(2), 2953-2967.

[97] Xie H. L.; Liang W. J.; Qin Y. S.; Lai X. J.; Li K. Q.; Su X. J.; Wu Y. H.; Wu W. J. Layered Heterostructure Enable Sensitive Thermosensation and High Fire Safety of Flexible Organic Thermoelectric Nanocoating. *Chem. Eng. J.* **2024**, 481, 148795.

[98] Ren P.; Li J.; Zhang X.; Yang X. Highly efficient solar water evaporation of TiO_2_@TiN hyperbranched nanowires-carbonized wood hierarchical photothermal conversion material. *Mater. Today Energy* **2020**, 18, 100546.

[99] Xing C.; Li Z.; Zhang S.; Bang J.; Xie Z.; Zhang H.; Peng Z. Phase Inversion-Based foam hydrogels for highly efficient Solar-Powered interfacial desalination. *Chem. Eng. J.* **2023**, 464, 142409.

[100] Zhu P.; Yu Z.; Sun H.; Zheng D.; Zheng Y.; Qian Y.; Wei Y.; Lee J.; Srebnik S.; Chen W.; Chen G.; Jiang F. 3D Printed Cellulose Nanofiber Aerogel Scaffold with Hierarchical Porous Structures for Fast Solar-Driven Atmospheric Water Harvesting. *Adv. Mater.* **2024**, 36(1), 2306653.

[101] Li W.; Tian X.; Li X.; Han S.; Li C.; Zhai X.-Z.; Kangc Y.; Yu Z.-Z. Ultrahigh solar steam generation rate of a vertically aligned reduced graphene oxide foam realized by dynamic compression. *J. Mater. Chem. A* **2021**, 9, 14859–14867.
